# Structural, electronic and thermodynamic properties of triatomic borate-terminated MXene surfaces

**DOI:** 10.1038/s41598-025-17851-z

**Published:** 2025-09-12

**Authors:** Guilherme Ribeiro Portugal, Johanna Rosen

**Affiliations:** Materials Design, Department of Physics, Chemistry and Biology (IFM), Linköping University, 58183, Linköping, Sweden

**Keywords:** MXenes, Density function theory, 2D Materials, Surface chemistry, Materials science, Theory and computation

## Abstract

MXenes, a rapidly growing family of two-dimensional carbides and nitrides, have attracted attention for their high electrical conductivity and highly tunable surface chemistry. The recent synthesis of MXenes featuring triatomic borate (BO$_{2}$) terminations via a molten route further expanded the range of achievable surface functionalities. Here, we employ density functional theory calculations to systematically investigate a selection of BO$_{2}$-terminated MXenes, including Ti$_{2}$N, Ti$_{2}$C, V$_{2}$C, Nb$_{2}$C, Ta$_{2}$C, Ti$_{3}$C$_{2}$, Ti$_{4}$N$_{3}$, Ti$_{4}$C$_{3}$, V$_{4}$C$_{3}$, Nb$_{4}$C$_{3}$, and Ta$_{4}$C$_{3}$. Our calculations reveal that such BO$_{2}$ polyanionic terminations significantly distort the MXene lattice, increasing the thickness of each M$_{n+1}$X$_{n}$ layer compared to the corresponding parent MAX phases. These structural changes are accompanied by pronounced near-surface charge transfer, indicative of strong bonding interactions between the MXene and BO$_{2}$ functional groups. Electronic structure analysis further demonstrates that surface BO$_{2}$ units introduce additional electronic states near the Fermi level, potentially enhancing transport properties relative to Cl-terminated MXenes. Thermodynamic modeling confirms that triatomic borate terminations are energetically favorable under realistic experimental conditions, explaining why these groups can dominate over chlorine terminations during the reported synthesis route. Collectively, our results elucidate how borate functionalization reshapes the structural and electronic properties of MXenes, offering valuable insights into the strategic engineering of advanced two-dimensional materials tailored for multifunctional applications.

## Introduction

MXenes, a family of two-dimensional (2D) carbides and nitrides derived from layered MAX phases, have become the focus of intense research due to their unique combination of high conductivity, robust mechanical strength, and flexible surface chemistry^1–3^. They are described by the general formula M$_{n+1}$X$_{n}$T$_{x}$ (where M is usually an early transition metal, X is C and/or N, and *n* = 1–4), and exhibit rich chemical versatility through tunable surface terminations (T$_{x}$), commonly including O, OH, and F groups, acquired during synthesis and processing^4^. These surface functional groups play a crucial role in shaping the physicochemical properties of MXenes, enabling their potential use in a wide range of applications, including energy storage^5^, electrocatalysis^6^, optoelectronics^7^, water purification^8^, and electromagnetic shielding^9^.

Early MXene research and synthesis strategies predominantly yielded simpler monoatomic surface terminations, typically introduced during the selective etching of MAX phases with hydrofluoric acid (HF). Lately, techniques like chemical vapor deposition (CVD)^10^ or molten salt-based selective etching^11^ have enabled the incorporation of halogens, chalcogens, and organic amines as surface terminations, expanding the synthetic toolbox for MXenes. Meanwhile, advances in polyatomic surface functional groups may be the path to an even richer landscape of terminations and properties. Recently, metaborate anions (BO$_{2}^{-}$) have been successfully introduced as MXene surface terminations via a flux-assisted eutectic molten salt etching approach in the presence of borax (Na$_{2}$B$_{4}$O$_7\cdot$10H$_{2}$O)^12,13^. In this case, CuCl$_{2}$ selectively removes the A-site element from the MAX (Ti$_{3}$AlC$_{2}$, Nb$_{2}$AlC, Ti$_{3}$AlCN, and TiNbAlC) phase, while borax is thermally decomposed and generates triatomic borate polyanions. Such BO$_{2}^{-}$ anions cap the MXene surface forming stable O–B–O bonds, providing BO$_{2}$-terminated MXenes with exceptional oxidative stability, boosted electrical conductivity, and enhanced ion-storage capacity. Unlike monoatomic surface terminations, metaborate functional groups can bridge multiple adjacent metal sites, facilitating extended electron delocalization and enhancing charge transport properties^13^.

A comprehensive understanding of how BO$_{2}$ terminations adsorb onto the MXene surface, thereby modifying its structural and electronic properties, as well as why they are energetically favored over competing chlorine (Cl) terminations under the reported synthesis conditions remains incomplete. Although experimental evidence confirms the successful production of BO$_{2}$-terminated MXenes, the interplay between preferred adsorption sites, lattice distortions, charge-transfer mechanisms, and other key properties has not yet been explored. Addressing these gaps is essential for the rational design of new 2D MXene-based materials, particularly for applications where surface chemistry dictates performance, including electronic devices, energy storage, and catalysis^14,15^.

Here, we present a systematic density functional theory (DFT) investigation of BO$_{2}$-terminated MXenes. After carefully designing our MXene models, the structural analysis revealed that BO$_{2}$ terminations induce modest structural distortions and promote thicker MXenes frameworks relative to Cl-terminated analogues and their respective parent MAX phases. Moreover, we explored how these functional groups reshape the MXene electronic structure by redistributing surface-related charge density and enhancing the density of states near the Fermi level in comparison to Cl-terminated MXenes. Finally, by mapping the adsorption free energy landscapes, we elucidate why BO$_{2}$-terminated surfaces preferentially emerge during the reported synthesis protocol despite the presence of competing chlorine species. This multifaceted investigation highlights the versatility of BO$_{2}$-functionalized MXenes, demonstrating their benefit as a platform for tunable surface chemistry. More broadly, these insights pave the way for the controlled synthesis of novel multicomponent surface terminations, unlocking new possibilities for MXenes with unprecedented chemical diversity and expanded application potential.

## Methods

### DFT calculations

The equilibrium structure of all simulated MXenes were determined using ab initio DFT calculations performed with the Vienna Ab Initio Simulation Package (VASP)^16–18^. The projector-augmented wave (PAW) method^19,20^ was employed to model electron-ion interactions, with a plane-wave basis set energy cutoff of 520 eV. During structural optimization, the atomic positions were iteratively adjusted until the Hellmann–Feynman forces acting on each atom were reduced to less than 10$^{-2}$ eV/Å. The Brillouin zone was sampled using a 11 $\times$ 11 $\times$ 1 **k**-point mesh and convergence of the electronic self-consistent loop was achieved when the total energy difference between successive iterations was below 10$^{-7}$ eV per atom.

For the bulk MAX phases, the exchange-correlation effects were treated using the generalized gradient approximation (GGA) based on the Perdew–Burke–Ernzerhof (PBE) functional^21^. In contrast, for both multilayered and monolayered MXenes, the revised van der Waals density functional (rev-vdW-DF2)^22^ was used to account for non-local dispersion interactions. Semilocal functionals such as PBE are known to systematically underestimate semiconductor band gaps because of residual self-interaction errors inherent to these functionals. Screened hybrid functionals (e.g., HSE06)^23^ and more developed approaches as self-interaction–corrected schemes^24^ largely remedy this deficiency, yet they are significantly more computationally demanding and can overestimate bandwidths or introduce nonphysical features in metals^25^. All BO$_2$-terminated MXenes examined in this study are metallic, and the observables of interest are already well described at the semilocal level. To treat the interlayer dispersion forces inherent to these van der Waals materials, we employed the revised vdW-DF2 non-local correlation functional. The resulting exchange-correlation framework therefore strikes an optimal balance between accuracy and computational efficiency for our metallic structures, and further implementation of hybrid functionals or other methodologies would not alter the qualitative conclusions reported herein.

**Fig. 1:**
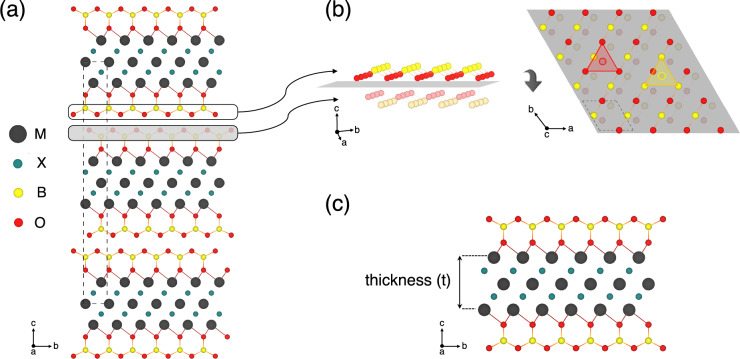
Structural models of BO$_{2}$-terminated MXenes. (**a**) Representative multilayered M$_{3}$X$_{2}$(BO$_{2}$)$_{2}$ structure with a misaligned stacking sequence along the *c*-axis. (**b**) Schematic of the misaligned stacking configuration between two adjacent M$_{3}$X$_{2}$(BO$_{2}$)$_{2}$ sheets. The left panel highlights the four surface-lying atomic layers displayed in the right panel, separated by a gray (001) plane. Atoms above the gray plane appear brighter, while those below are darker. Each B or O atom of one MXene sheet aligns centrally within the triangular arrangement of their counterparts in the adjacent layer. (**c**) Representative single sheet M$_{3}$X$_{2}$(BO$_{2}$)$_{2}$ structure. The layer thickness (t) is defined as the vertical distance between opposing outermost M (metal) atomic planes. The color scheme (M = dark gray, X = teal, B = yellow, O = red) is consistent across all panels. For clarity, both the monolayered and the multilayered structure present BO$_{2}$ terminations at the FCC site.

The design of BO$_{2}$-terminated MXenes was based on the experimental report by Li and colleagues in Ref.^13^. Beyond their inherently intriguing multilayered architecture, the fact that delamination has been experimentally demonstrated exclusively for Ti$_{3}$C$_{2}$(BO$_{2}$)$_{2}$ further motivated our investigation into other multilayered MXenes structures. From a broad range of M$_{n+1}$AlX$_{n}$ parent MAX phases, we selected Ti$_{2}$AlN, Ti$_{2}$AlC, V$_{2}$AlC, Nb$_{2}$AlC, Ta$_{2}$AlC, Ti$_{3}$AlC$_{2}$, Ti$_{4}$AlN$_{3}$, Ti$_{4}$AlC$_{3}$, V$_{4}$AlC$_{3}$, Nb$_{4}$AlC$_{3}$, and Ta$_{4}$AlC$_{3}$, prioritizing those with established experimental viability for 2D MXene synthesis. The study comprised full structural relaxation of the selected parent MAX phases, all exhibiting ($P6_{3}/mmc$) symmetry, using 1 $\times$ 1 $\times$ 1 supercells containing 8, 12, and 16 atoms for *n* = 1, 2, and 3, respectively. Further, aluminum (Al) atoms were removed from the unit cell, and termination species were introduced to build fully terminated multilayered MXenes with the general formula M$_{n+1}$X$_{n}$T$_{2}$ (T = Cl, BO$_{2}$), as illustrated in Fig. 1a. The resulting supercells consisted of (10) 18, (14) 22, and (18) 26 atoms for (Cl-) BO$_{2}$-terminated MXenes when *n* = 1, 2, and 3, respectively. For clarity, MXenes functionalized with BO$_{2}$ or Cl groups are designated as BO$_{2}$-MXenes or Cl-MXenes throughout this work. The abbreviated M$_{n+1}$X$_{n}$ notation (e.g., Ti$_{2}$C, Ti$_{3}$C$_{2}$) explicitly represents fully terminated M$_{n+1}$X$_{n}$T$_{x}$ systems (T = Cl or BO$_{2}$); it is critical to note that these do not correspond to bare MXenes. Terminations are explicitly stated in all cases to avoid ambiguity.

Even though the primary focus was on BO$_{2}$-MXenes, Cl-terminated MXenes were considered to enable comparative analyses, particularly regarding competition between those termination species which stand as the most probable ones according to the reported synthesis protocol. All simulated MXenes were modeled with fully-terminated surfaces, consistent with the experimental report where borate-terminated MXenes were synthesized with complete functionalization^13^. Given the availability of different adsorption sites for the surface terminations, three configurations were considered: the top site, where termination species are located directly above the surface M atoms; the hollow HCP site, corresponding to terminations above X atoms; and the hollow FCC site, where the functional groups are positioned over M atoms of the inner layers (Fig. S1).

In multilayered MXenes, the stacking of layers can follow various sequences. Based on recent findings^26,27^ and the experimentally reported data^13^, we considered the layers to be stacked according to a *misaligned* stacking configuration. In this set up, the terminations of adjacent stacked layers do not have the same *x* and *y* coordinates, and are positioned so that each B or O atom is centered in the triangular arrangement of their respective counterparts in the adjacent surface layer (Fig. 1b). After fully relaxing all multilayered MXenes, one layer was removed from each structure to form their respective monolayered MXene (Fig. 1c). This approach ensured a thick vacuum layer of at least 15 Å  in between periodic monolayer images to avoid spurious self-interaction. The atomic positions were then re-optimized while retaining the same supercell volume and shape. Convergence tests indicated that the relaxation of multilayered structures adequately captured the lattice parameter relaxation of monolayered MXenes, with energy differences smaller than 1 meV/atom.

### Charge analysis

In addition to structural optimizations and energy calculations, we performed Bader charge analysis^28,29^ to quantify net electron transfer among individual species in each system. Moreover, using VASPKIT code for post-processing of the VASP calculated data^30^, we computed the charge density difference (CDD) to visualize the electron transfer upon adsorption via $\Delta \rho (\textbf{r}) = \rho _\mathrm {MXene+T_x}(\textbf{r}) - \rho _\textrm{MXene}(\textbf{r}) - \rho _\mathrm {T_x}(\textbf{r})$, where $\rho _\mathrm {MXene+T_x}(\textbf{r})$ is the total charge density of the terminated MXene, $\rho _\textrm{MXene}(\textbf{r})$ is the charge density of the bare MXene, and $\rho _\mathrm {T_x}(\textbf{r})$ is the charge density of the isolated termination group in the exact same atomic coordinates, all of them in e/Å$^{3}$. For visual clarity, we also evaluated the planar-averaged charge density difference (PA-CDD) along the out-of-plane (*z*-direction) to track charge redistribution across the layers. The PA-CDD is obtained by integrating the CDD over the in-plane (*x-y*) directions through $\Delta \rho (z) = \frac{1}{A} \int \Delta \rho (x,y,z)dxdy$, where *A* is the cross-sectional area of the unit cell. This approach highlights spatial regions of electron accumulation or depletion upon surface termination adsorption.

## Results

### Structural properties

We begin by briefly discussing the preferred adsorption sites of BO$_{2}$ terminations for each simulated MXene, a crucial factor in determining their stability and overall properties. As displayed in Table 1, the FCC site is consistently the energetically preferred adsorption site in M$_{2}$X and M$_{3}$X$_{2}$ BO$_{2}$-MXenes. This is also the case for Cl-terminated MXenes (Table S1). However, in the higher-order M$_{4}$X$_{3}$ family, the FCC site is the preferred adsorption site only for Ti-based MXenes. Changing the metal to V, Nb, or Ta shifts the energetic preference toward the HCP site, regardless of the surface functional group (BO$_{2}$ or Cl). A potential discrepancy arises when comparing our results with the experimentally reported Ti$_{3}$C$_{2}$(BO$_{2}$)$_{2}$^13^, which was suggested to have BO$_{2}$ terminations at the HCP site. In contrast, our calculations indicate that the FCC site is approximately 25 meV/atom more energetically favorable than the HCP. Therefore, the most energetically stable sites were adopted as references ensuring that subsequent discussions on energetics and structure-property relationships are based on the lowest energy configuration for each MXene. The relaxed structures of all simulated MXenes are provided in Figs. S2 and S3.

**Table 1:** Optimized lattice parameters of multilayered BO$_{2}$-terminated MXenes.

MXene	BO_2_ site	$a=b$ (Å)	*c* (Å)	$\alpha \ (^{\circ })$	$\beta \ (^{\circ })$	$\gamma \ (^{\circ })$
Ti_2_N	FCC	3.04	23.55	84.36	95.64	129.68
Ti_2_C	FCC	3.04	23.46	85.36	94.64	128.61
V_2_C	FCC	3.00	23.45	85.41	94.59	129.74
Nb_2_C	FCC	3.17	24.45	84.18	95.82	130.88
Ta_2_C	FCC	3.14	24.50	84.84	95.16	130.36
Ti_3_C_2_	FCC	3.05	28.44	86.72	93.28	127.01
Ti_4_N_3_	FCC	3.00	33.39	87.22	92.77	126.15
Ti_4_C_3_	FCC	3.05	33.42	88.01	91.99	125.85
V_4_C_3_	HCP	2.89	33.46	90.35	89.65	124.85
Nb_4_C_3_	HCP	3.11	35.00	89.68	90.32	125.61
Ta_4_C_3_	HCP	3.08	34.88	89.67	90.33	125.08

For each compound, the energetically preferred BO$_{2}$ adsorption site (FCC or HCP) is reported together with their respective in-plane ($a=b$) and out-of-plane (*c*) lattice constants, and the three lattice angles $\alpha$, $\beta$, and $\gamma$.

**Fig. 2:**
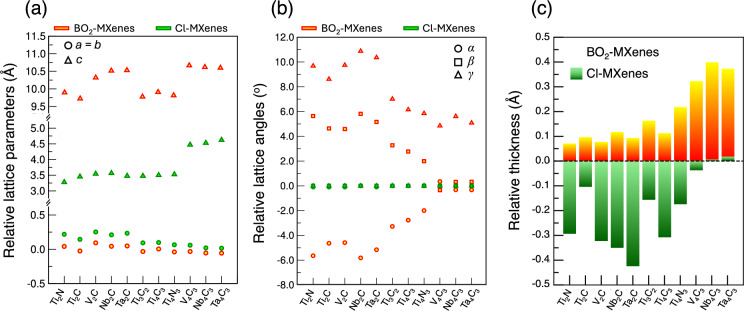
Structural relaxation of multilayered BO$_{2}$- and Cl-terminated MXenes relative to their parent MAX phases. (**a**) Relative differences in the in-plane (*a* = *b*) and out-of-plane (*c*) lattice parameters; (**b**) Relative differences in the in-plane ($\alpha$, $\beta$) and out-of-plane ($\gamma$) lattice angles; (**c**) Relative thickness differences of each MXene, schematically defined in Fig. 1c, highlighting the contrasting structural effects imposed by BO$_{2}$ and Cl terminations. Red and yellow markers indicate properties of BO$_{2}$-MXenes, while green markers correspond to Cl-MXenes.

An overview of the structural relaxation induced by BO$_{2}$ terminations in terms of lattice parameters (LP) and angles is also given in Table 1. Importantly, the LPs obtained for Nb$_{2}$C(BO$_{2}$)$_{2}$ and Ti$_{3}$C$_{2}$(BO$_{2}$)$_{2}$ are overestimated by 3.4–4.6% relative to experimental values^13^. While these deviations slightly exceed the typical margin observed for rev-vdW-DF2 functionals^31,32^, most likely due to the inherent complexity of modeling the exact the O–B–O stacking obtained experimentally, they remain within acceptable tolerances for such systems. Nonetheless, our computational model provide a robust approximation of idealized defect-free systems, providing foundational understanding of their structural behavior.

While certain trends are apparent across different MXene compositions and orders, notable structural differences emerge when comparing the effects of the two competing termination species, BO$_{2}$ and Cl, on MXene relaxation. The LPs, angles, and thicknesses relative to each respective parent MAX phase are displayed Fig. 2 (values in Tables S2–S4). Regarding lattice parameters (Fig. 2a), Cl-terminated MXenes consistently exhibit positive and larger in-plane expansion compared to their BO$_{2}$-terminated counterparts, indicating a more pronounced structural relaxation within the basal plane. Conversely, BO$_{2}$-MXenes demonstrate substantially larger out-of-plane relaxation (*c*-LP) in comparison to Cl-MXenes, which can be attributed to the vertical pillaring effect introduced by triatomic BO$_{2}$ surface groups. Furthermore, after capping the MXene surface with M–O bonds, BO$_{2}$ terminations adopt a unique O–B–O configuration, with boron atoms threefold-coordinated by oxygen. The thickness of the BO$_{2}$ termination layer, defined as the vertical distance between the outermost metal layer and terminal oxygen atoms (Table S5), remains within the narrow range of approximately 3.3–3.5 Å, closely matching the reported values for BO$_{2}$-terminated Ti$_{3}$C$_{2}$ and Nb$_{2}$C MXenes^13^.

The structural relaxation induced by BO$_{2}$ terminations is further evident by their impact on lattice angles (Fig. 2b). MXenes functionalized with Cl terminations retain near-perfect hexagonal symmetry, with $\alpha \approx \beta \approx$ 90$^{\circ }$ and $\gamma \approx$ 120$^{\circ }$, exhibiting negligible deviations from their parent MAX phases. In turn, BO$_{2}$ terminations drive significant angular distortions. Interestingly, $\alpha$ and $\beta$ display an isotropic compensation mechanism: $\alpha$ decreases below 90$^{\circ }$ whereas $\beta$ exceeds 90$^{\circ }$ by the exact same amount, preserving an average of 90$^{\circ }$. Lower-order BO$_{2}$-M$_{2}$X structures exhibit larger angular deviations than BO$_{2}$-M$_{4}$X$_{3}$ systems, as the structural flexibility of the former enables greater relaxation. This is also mirrored in $\gamma$, which falls above 120$^{\circ }$ in all cases but with more pronounced distortions in lower-order MXenes. The enhanced structural rigidity introduced by additional M–X layers partially suppresses angular distortions, yielding a lattice structure more similar to the typical hexagonal symmetry of the parent MAX phases. Although these distortions are challenging to characterize experimentally, multiple studies have nonetheless documented that distinct surface terminations and ion intercalation/adsorption may lead to pronounced lattice deviations in MXenes, which lose the typical *P6*_3_*/mmc* symmetry of the parent MAX phase^33–36^.

**Fig. 3:**
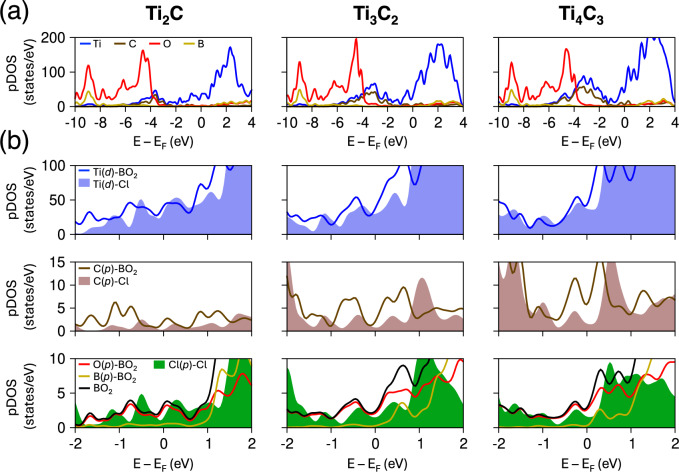
Projected density of states (pDOS) for Ti$_{2}$C (left panels), Ti$_{3}$C$_{2}$ (central panels), and Ti$_{4}$C$_{3}$ (right panels), with both BO$_{2}$ and Cl terminations. (**a**) pDOS for BO$_{2}$-terminated MXenes. (**b**) Close-up comparison of the pDOS for each atomic species in BO$_{2}$- (solid lines) vs. Cl-terminated (shaded area) MXenes. The black line (bottom panels) shows the total DOS of BO$_{2}$ units. In both (**a,b**), the *y*-axis scale is consistent across all three plots in the same row.

Another relevant difference emerges upon analyzing the thickness (schematically defined in Fig. 1c) of each MXene M–X framework following surface functionalization. Figure 2c shows the relative thickness changes for each MXene compared to their respective parent MAX phase. Negative relative thickness (contraction) is induced by Cl-terminated MXenes, reflecting their exclusively positive and more prominent in-plane LPs relaxation that increases *a* and *b*. On the other hand, BO$_{2}$-terminated MXenes always display positive relative thickness (swelling), arising from comparatively smaller in-plane LPs changes that hence require greater out-of-plane relaxation to stabilize the system. It is noted that MXenes with negative relative in-plane LPs changes, namely Ti$_{2}$C, Ti$_{3}$C$_{2}$, Ti$_{4}$N$_{3}$, V$_{4}$C$_{3}$, Nb$_{4}$C$_{3}$, and Ta$_{4}$C$_{3}$, display the highest relative change in thickness, given the natural compressive strain imposed by such a relaxation. Additionally, higher-order BO$_{2}$-MXenes exhibit even larger thickness swelling than lower-order ones as a consequence of their reduced in-plane flexibility both in terms of lattice constants (*a* = *b*) and angles ($\alpha$ and $\beta$), which intensifies the out-of-plane relaxation.

The contrasting structural responses observed between monoatomic Cl and triatomic BO$_{2}$ terminations are fundamentally linked to their distinct bonding and charge redistribution behavior. While Cl terminations predominantly induce basal-plane expansions without significantly affecting the overall MXene structure or out-of-plane lattice constant, BO$_{2}$ terminations exhibit complex structural rearrangements, lattice-angle distortions, and vertical swelling. Such distinct structural changes, especially in BO$_{2}$-MXenes, are intimately related to their electronic structure, explored in the following section.

### Electronic properties

The electronic structure of MXenes originates mainly from metal *d*-orbitals interacting with light-element *p*-orbitals from both core layers and surface terminations. The extent and nature of this orbital hybridization near the Fermi level (E$_\text {F}$) governs and defines transport properties, band alignment, and other fundamental electronic properties. To establish a direct comparison between BO$_{2}$- and Cl-terminated MXenes, we first investigate the projected density of states (pDOS) of their multilayered structures. The pDOS of monolayered MXenes (not shown here) remains nearly unchanged relative to their multilayered system, reinforcing that interlayer interactions are predominantly governed by weak van der Waals forces rather than significant electronic coupling.

Figure 3 presents the pDOS for multilayered Ti$_{2}$CT$_{2}$ (left panels), Ti$_{3}$C$_{2}$T$_{2}$ (central panels), and Ti$_{4}$C$_{3}$T$_{2}$ (right panels) MXenes, where T = BO$_{2}$ and Cl. The broad energy and pDOS range displayed in Fig. 3a reveals distinct electronic states well below the Fermi level (defined at 0 eV), attributed to strong and stable Ti–C, Ti–O, and B–O bonding. The E$_\text {F}$ vicinity, however, is essentially dominated by Ti *d*-orbitals, confirming that electronic conduction in these MXenes is essentially associated to the transition-metal sublattice. This feature is observed across all BO$_{2}$-terminated MXenes (see Figs. S4–S6), where the dominant metal-centered states near E$_\text {F}$ suggests a robust metallic character.

A direct comparison between BO$_{2}$- and Cl-terminated MXenes reveals that BO$_{2}$ functionalization enhances the contributions of both Ti *d* and C *p* states near the Fermi level (Fig. 3b). Isolating the surface termination contributions, the *p*-orbitals from O and B exhibit a significantly higher density of states around E$_\text {F}$ compared to Cl *p*. Considering the whole BO$_{2}$ unit (black curve), the overall density of states is clearly increased relative to Cl terminations. As shown in Figs. S4–S6, this is a general trend for all investigated MXenes. From a functional perspective, an enhanced pDOS near E$_\text {F}$ indicates a greater number of accessible electronic states, which can translate into higher carrier mobility and improved charge-transport properties.

**Fig. 4:**
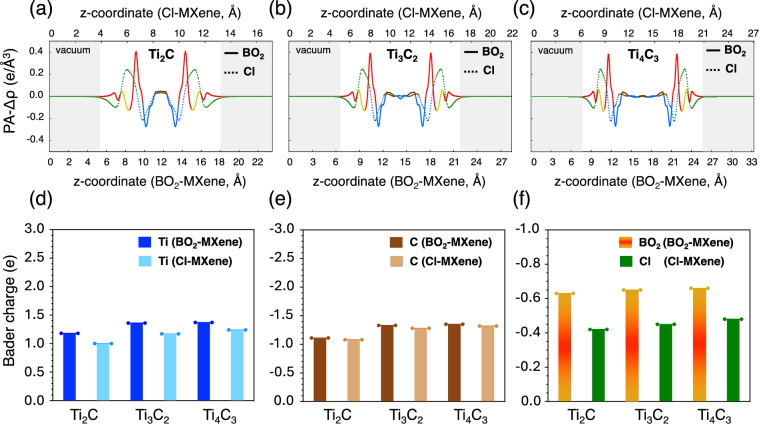
Charge analysis for monolayered Ti-based MXenes. (**a–c**) Planar-averaged charge density difference (PA-CDD) along the out-of-plane (*z*) direction, comparing BO$_{2}$ (solid line) and Cl (dotted line) terminations. Positive PA-CDD indicates electron accumulation, whereas negative values reflect electron depletion, both relative to the combined reference state of bare MXene and isolated termination species. Colors along the lines change progressively according to distinct atomic planes encountered along the *z*-coordinate: green for Cl, blue for Ti, red for O, golden for B, and brown for C. The vacuum region is shaded in gray. (**d–f**) Corresponding Bader charges for Ti, C, and Cl/BO$_{2}$ species, respectively. The higher positive charge of Ti and more negative charge of O/B in BO$_{2}$-MXenes reflect stronger electron transfer compared to Cl-MXenes.

In order to understand how BO$_{2}$ terminations influence the charge distribution in the proposed MXenes, we looked into the electronic charge density rearrangement upon BO$_{2}$ or Cl adsorption. Figure 4a–c exhibits the planar-averaged charge density difference (PA-CDD) along the out-of-plane direction for monolayered Ti-based MXenes. Positive peaks correspond to electron accumulation in the terminated MXene relative to the reference state (bare MXene + isolated termination group), while negative peaks indicate electron depletion. These features reflect charge redistribution arising from bonding interactions between the MXene and surface terminations. BO$_{2}$-MXenes (solid lines) consistently show significant peaks on the termination layers, indicating a substantial charge transfer from Ti layers toward BO$_{2}$ sites. On the other hand, Cl-MXenes display less pronounced peaks, pointing to a smaller net electron transfer to the halogen atoms. The three-dimensional CDD plots (Fig. S7) further confirm that oxygen atoms in BO$_{2}$ terminations draw electrons more effectively from Ti inner layers than Cl ones. The PA-CDD data in Fig. S8 shows that this trend holds across all investigated MXenes.

As a complement to the PA-CDD analysis, the Bader charge data in Fig. 4 (d-f) provide a more quantitative view of the charge distribution among Ti, C, and the terminating species for Ti-based MXenes (see also Tables S6–S10). In all cases, metal atoms in BO$_{2}$-terminated MXenes (in this case, Ti) carry a higher positive charge than those in Cl-terminated MXenes, reflecting the stronger potential that triatomic borate groups have to attract electrons from the bare MXene surface. Even though the Bader charge on C atoms remains largely unchanged, BO$_{2}$ functional groups exhibit a significantly higher localized negative charge compared to Cl terminations, reflecting stronger bonding interactions between BO$_{2}$ terminations and the MXene sheet.

In summary, the electronic structure analysis indicate that BO$_{2}$ terminations induce greater net charge transfer than Cl, leading to more polarized bonds between the metal M and functional groups. This higher charge transfer suggests a stronger interaction between BO$_{2}$ groups and the MXene surface, implying that such terminations may preferentially attach when both BO$_{2}$ and Cl functional groups are present during synthesis. Nevertheless, charge transfer alone is not a definitive predictor of termination stability. Instead, determining which termination ultimately prevails on the MXene surface requires evaluating adsorption free energies under experimentally relevant chemical potential conditions, a topic addressed in the following section.

### Thermodynamics of BO$_{2}$ functionalization

The tunable surface chemistry of MXenes depend strictly on the thermodynamic stability of their functional groups when anchored to the surface, which governs synthesis feasibility and termination selectivity under experimental conditions. To better understand the driving force behind the metaborate functionalization, as well as its competition with chlorine terminations, we employed a rigorous thermodynamic approach centered on adsorption free energy ($\Delta \text {H}_{ads}^{\text {T}_{x}}$). This metric quantifies the energetic favorability of adsorbing a termination group T$_{x}$ (e.g., (BO$_{2}$)$_{2}$, Cl$_{2}$) onto a bare MXene surface, with the latter serving as the reference state. The adsorption free energy is defined as

1 $$\begin{aligned} \Delta \text {H}_{ads}^{\text {T}_{x}} = \frac{\left[ \text {H}(\text {M}_{n+1}\text {X}_{n}\text {T}_{x}) - \text {H}(\text {M}_{n+1}\text {X}_{n}) \right] - \sum _{i=1}x_{i}\mu _{i}}{x}, \end{aligned}$$

where H($\text {M}_{n+1}\text {X}_{n}\text {T}_{x}$) and $\text {H}(\text {M}_{n+1}\text {X}_{n})$ are the total energies of the terminated and bare MXenes, respectively, *x* is the number of terminations (here *x* = 2 for fully terminated surfaces), and $\mu$ the chemical potential of the relevant termination species. A negative $\Delta \text {H}_{ads}^{\text {T}_{x}}$ indicates spontaneous adsorption, whereas a positive value suggests that surface functionalization is not energetically favorable. In this framework, we systematically evaluate whether borate groups stabilize MXenes relative to Cl terminations, providing critical insights into the equilibrium conditions required for their experimental realization.

To illustrate the thermodynamic modeling approach applied for all MXenes, we take Ti$_{3}$C$_{2}$, the benchmark MXene and, to date, the only member reliably synthesized with BO$_{2}$ terminations, as a representative example. In order to plot $\Delta \text {H}_{ads}^{\text {T}_{x}}$ for BO$_{2}$ and Cl terminations, we must first determine their accessible chemical potentials ranges. For Cl-MXenes, the chemical potential of chlorine ($\mu _{\text {Cl}}$) is straightforwardly defined and constrained (*vide infra*). In turn, given the instability and reactivity of metaborate ions, defining $\mu _{\text {BO}_{2}}$ requires a more careful approach. Since BO$_{2}^{-}$ ions originate from NaBO$_{2}$, generated during the decomposition of borax, they are likely to adsorb onto the MXene surface as intact units. Therefore, $\mu _{\text {BO}_{2}}$ was constructed accordingly (see Supporting Information for details).

The evaluation Cl or BO$_{2}$ adsorption starts with establishing the lower limits for both $\mu _{\text {Cl}}$ and $\mu _{\text {BO}_{2}}$. These limits correspond to the conditions where $\Delta \text {H}_{ads}^{\text {T}_{x}}$ becomes zero, i.e., the threshold where adsorption ceases to be thermodynamically favorable. For Cl terminations, this occurs when $\mu _{\text {Cl}}$ = −3.82 eV, whereas for BO$_{2}$ it occurs when $\mu _{\text {BO}_{2}}$ = −21.68 eV. If the chemical potentials lie below these limits, the formation of Cl- or BO$_{2}$-terminated MXenes becomes energetically unfavorable, defining the least favorable conditions under which adsorption remains thermodynamically preferred and ensuring a balanced comparison between the two functional groups. Since the focus was on the competitive regime where both Cl and BO$_{2}$ functionalities can coexist, we adopted such values as the respective lower limits for Ti$_{3}$C$_{2}$T$_{2}$.

**Fig. 5:**
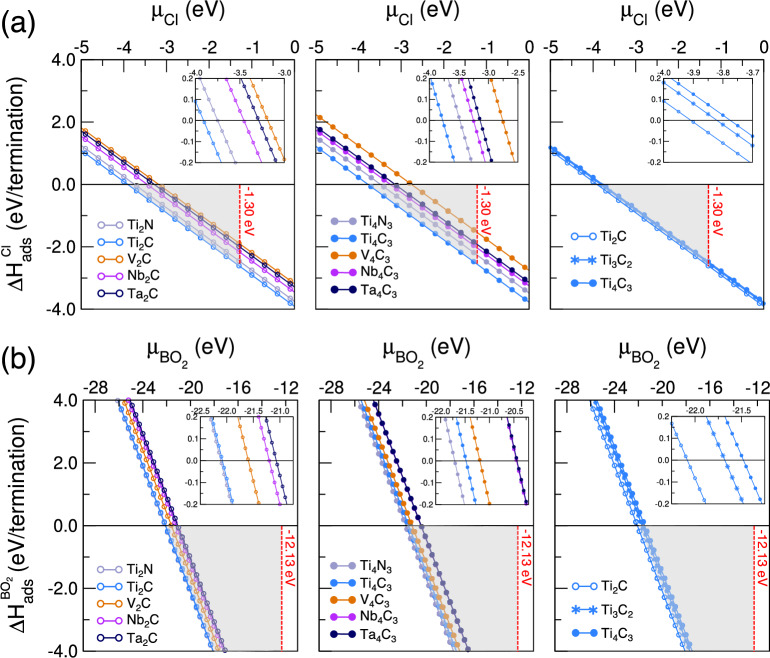
Adsorption free energy ($\Delta \text {H}_{ads}^{\text {T}_{x}}$) dependence on the chemical potentials $\mu _{\text {Cl}}$ (**a**) and $\mu _{\text {BO}_{2}}$ (**b**) for Cl- and BO$_{2}$-terminated MXenes. Left, central, and right panels show the adsorption behavior for M$_{2}$X, M$_{4}$X$_{3}$, and Ti-based MXenes, respectively. Gray-shaded regions correspond to the stability ranges of Cl and BO$_{2}$ terminations upon chemical potentials constrains. Red vertical lines mark the upper limits for $\mu _{\text {Cl}}$ and $\mu _{\text {BO}_{2}}$ potentials.

Next, we establish the upper boundaries for such chemical potentials by considering the stability of the precursors for each functional group. In Cl-MXenes, Cl$^{-}$ terminations originate from CuCl$_{2}$, which constrains $\mu _{\text {Cl}}$ according to its equilibrium (see Supporting Information for thermodynamic derivations). As result, $\mu _{\text {Cl}}$ cannot exceed −1.30 eV under the reported conditions (Fig. S11). Meanwhile, BO$_{2}^{-}$ ions are derived from NaBO$_{2}$ upon borax decomposition, constraining the upper limit for $\mu _{\text {BO}_{2}}$ to −12.13 eV (Fig. S12). Having defined these constraints, the full range of $\mu _{\text {Cl}}$ and $\mu _{\text {BO}_{2}}$ for Ti$_{3}$C$_{2}$ can be expressed as $-3.82 \; \text {eV} \le \mu _{\text {Cl}} \le -1.30 \; \text {eV}$ and $-21.68 \; \text {eV} \le \mu _{\text {BO}_{2}} \le -12.13 \; \text {eV}$ (Fig. S13). Having established the thermodynamic framework for BO$_{2}$ and Cl adsorption on Ti$_{3}$C$_{2}$, we then applied the same methodology to all remaining MXene compositions under investigation. Provided that synthesis conditions remain similar (with only minor temperature adjustments), the upper limits for $\mu _{\text {Cl}}$ and $\mu _{\text {BO}_{2}}$ remain unchanged. Still, their lower limits shift according to the specific $\Delta \text {H}_{ads}^{\text {T}_{x}}$ for each particular MXene. Table S11 compiles the resulting chemical potential ranges for all investigated structures.

Figure 5 displays the adsorption free energy dependence on $\mu _{\text {Cl}}$ in panel (a) and $\mu _{\text {BO}_{2}}$ in panel (b). The gray-shaded regions mark the chemical potential ranges achievable under the assumed experimental conditions. For a given termination species, M$_{2}$X systems consistently show more negative $\Delta \text {H}_{ads}^{\text {T}_{x}}$ than their M$_{4}$X$_{3}$ analogs, suggesting a stronger driving force for termination adsorption in lower-order MXenes. This is evident in Ti-based MXenes (rightmost panels), where at fixed $\mu _{\text {Cl}}$ and $\mu _{\text {BO}_{2}}$ the adsorption free energy becomes less negative from Ti$_{2}$C to Ti$_{4}$C$_{3}$. Additionally, Ti-based MXenes exhibit the lowest $\Delta \text {H}_{ads}^{\text {T}_{x}}$ values and the broadest stability windows (see Table S9) among all MXenes, implying that even under poor Cl or BO$_{2}$ conditions, Ti$_{n+1}$C$_{n}$ phases still favor surface functionalization. Another trend is evident when comparing the critical chemical potential limits where $\Delta \text {H}_{ads}^{\text {T}_{x}}$ = 0 eV. For Cl-terminated MXenes (inset of Fig. 5a), the limits rank as Nb < Ta < V, whereas for BO$_{2}$-terminated MXenes (inset Fig. 5b), the order switches to V < Nb < Ta. This inversion reflects differences in covalency and charge transfer between halogen-metal versus oxygen-metal bonding, coupled with each metal’s unique *d*-electron occupancy and ionic radius.

Despite these specifics, the broader conclusion is that Cl and BO$_{2}$ functional groups strongly compete for adsorption within the examined chemical potential space. In general, BO$_{2}$ adsorption curves are found at more negative energies, indicating that sufficiently high $\mu _{\text {BO}_{2}}$ might render BO$_{2}$-terminated surfaces more stable than their Cl-terminated competitors. For instance, in an extremely Cl-rich environment, Ti$_{3}$C$_{2}$ exhibits $\Delta \text {H}_{ads}^{\text {Cl}}$ = −2.52 eV. To achieve the same adsorption free energy for BO$_{2}$-terminations, a $\mu _{\text {BO}_{2}}$ = −19.15 eV is required, which is about 7.0 eV lower than its reference upper limit (−12.13 eV). Under a slightly richer metaborate condition (e.g., $\mu _{\text {BO}_{2}}$ = −15.0 eV), the adsorption free energy for BO$_{2}$ shifts notably toward more negative values (in this case, $\Delta \text {H}_{ads}^{\text {BO}_{2}}$ = −6.68 eV). This demonstrates that $\Delta \text {H}_{ads}^{\text {T}_{x}}$ can be significantly lower for BO$_{2}$ units, thus making them more favorable than Cl terminations. Nonetheless, while BO$_{2}$ seems to prevail, the exact preference depends explicitly upon the choice of the metal M, the X element (C or N), and the experimentally accessible chemical potential ranges. These findings clarify how BO$_{2}$-terminated MXenes can form even under Cl-rich synthesis conditions, stressing the critical role of MXene composition and reaction environment on termination stability.

**Fig. 6:**
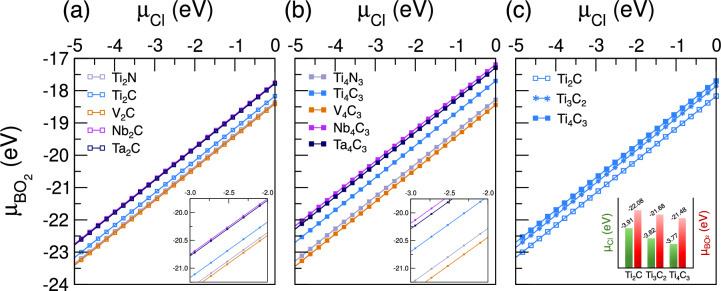
Adsorption free energy boundary ($\Delta \text {H}_{ads}^{\text {Cl}}$ = $\Delta \text {H}_{ads}^{\text {BO}_{2}}$) for different (**a**) M$_{2}$X, (**b**) M$_{4}$X$_{3}$, and (**c**) Ti-based MXenes dictated by the chemical potentials of their terminating functional groups. The inset bar chart in (**c**) displays the specific lower chemical potential limits for Ti-based MXenes, which increase (move towards richer Cl or BO$_{2}$ regions) when increasing the MXene family order.

Finally, Fig. 6 presents the adsorption free energy boundary where $\Delta \text {H}_{ads}^{\text {Cl}}$ = $\Delta \text {H}_{ads}^{\text {BO}_{2}}$. The region above (below) each sloped line favors BO$_{2}$ (Cl) adsorption. As expected, the position of each line depends both on the metal and the MXene order. In Fig. 6a, Ti$_{2}$C lies near Ti$_{2}$N, while V$_{2}$C, Nb$_{2}$C, and Ta$_{2}$C shift according to the metal. A similar trend is observed for higher-order MXenes in Fig. 6b, though in a somewhat different region of the $\mu _{\text {Cl}}$-$\mu _{\text {BO}_{2}}$ space. As the MXene order increases, the number of layers and the valence state of surface metal atoms change, altering the balance between Cl and BO$_{2}$ functionalization. This effect is more clearly visualized in Fig. 6c, where Ti-based MXenes of different orders are directly compared. The chemical potential threshold for each equilibrium line moves toward less negative values when moving from Ti$_{2}$C to Ti$_{3}$C$_{2}$ and then to Ti$_{4}$C$_{3}$. Hence, under the same $\mu _{\text {Cl}}$ constraints, achieving BO$_{2}$-terminated surfaces in higher-order MXenes demands richer $\mu _{\text {BO}_{2}}$ conditions. The inset, obtained by setting $\Delta \text {H}_{ads}^{\text {T}_{x}}$ = 0 eV, supports such a conclusion and confirms that the range in which Cl or BO$_{2}$ adsorption remains spontaneous grows narrower (smaller bars = richer conditions) from Ti$_{2}$C to Ti$_{4}$C$_{3}$, which reflects the reduced flexibility in the chemical potential space for thicker MXenes.

For comparison, an alternative approach reported in the literature for evaluating termination preferences was employed, which involves total energy summations of reaction products to infer thermodynamic stability of a given termination over the competing one (see Supporting Information). For instance, in the case of BO$_{2}$-terminated MXenes, such an approach revealed that the preference for BO$_{2}$ over Cl terminations arises from the relative energies of the final products in hypothetical surface-capping reactions (Eqs. S25–S28)^13^. To test this methodology, we applied the same summation approach to our Ti$_{3}$C$_{2}$ and Nb$_{2}$C MXene models (monolayered and multilayered configurations). Contrary to the adsorption free energy results and the experimentally observed dominance of BO$_{2}$ terminations, the calculations using this method consistently favored Cl-terminated MXenes across all systems (Table S12, Figs. S9, S10). For example, at the PBE level, the total energy difference for Ti$_{3}$C$_{2}$ indicated a 1.4 eV preference for Cl over BO$_{2}$ terminations, a trend mirrored in all evaluated MXenes. While these values align in magnitude with a prior report^13^, the opposite energetic preference highlights that comparing DFT total energies without properly accounting for chemical potentials and reference phases may not reflect the true thermodynamic driving forces for termination adsorption.

Therefore, while simplified reaction-based analyses offer intuitive insights, neglecting system-specific chemical environments may well lead to misinterpretation. In contrast, the adsorption free energy approach incorporating termination-dependent chemical potentials reliably reconciles computational predictions with experimental observations. By mapping stability boundaries in $\mu _{\text {Cl}}$–$\mu _{\text {BO}_{2}}$ space, this method clarifies how MXene composition (transition metal, stoichiometry) and synthesis conditions synergistically govern termination preferences. In this case, the experimentally realized BO$_{2}$-MXenes occupy distinct stability regions where $\mu _{\text {BO}_{2}}$ is sufficiently negative to offset Cl competition, even in a Cl-rich molten salt environment. These findings not only rationalize existing experimental achievements but also strengthen our ability to engineer MXene surface terminations, a critical factor in tailoring their properties for various applications.

## Discussion

We employed density functional theory to comprehensively explore the structural features, electronic behavior, and surface termination adsorption properties of BO$_{2}$-terminated MXenes, covering a range of compositions and stoichiometries (Ti$_{2}$N, Ti$_{2}$C, V$_{2}$C, Nb$_{2}$C, Ta$_{2}$C, Ti$_{3}$C$_{2}$, Ti$_{4}$N$_{3}$, Ti$_{4}$C$_{3}$, V$_{4}$C$_{3}$, Nb$_{4}$C$_{3}$, and Ta$_{4}$C$_{3}$). Our calculations revealed that triatomic BO$_{2}$ terminations induce significant but consistent lattice distortions and increase the thickness of MXenes layers relative to their parent MAX phases. Electronically, the introduction of BO$_{2}$ terminations generates additional electronic states near the Fermi level, potentially enhancing transport properties in such MXenes. Furthermore, BO$_{2}$-MXenes displayed considerably higher charge transfer upon termination adsorption compared to Cl-MXenes, an indicative of strong chemical interactions between the BO$_{2}$ groups and MXene surfaces. Our adsorption free-energy analyses further demonstrate that under experimentally relevant chemical potentials, BO$_{2}$ rich environments may well thermodynamically favor BO$_{2}$- over Cl-functionalized MXene surfaces, elucidating why the reported molten salt syntheses yielded only BO$_{2}$-terminated MXenes despite the presence of Cl salts as CuCl$_{2}$. A systematic extension of the present methodology to MXenes bearing the more common O-, F-, and OH- terminations, as well as to those with other polyatomic terminations, should be examined in greater depth. Future work could likewise examine how relevant ions and molecules adsorb on BO$_{2}$- and other polyatomic-terminated MXene surfaces, thereby clarifying the interplay between termination chemistry, interfacial interactions, and application-specific performance. Overall, this work establishes a fundamental understanding of how triatomic borate terminations influence the structural and electronic properties of different MXenes providing valuable thermodynamic insights and guidelines for strategically designing next-generation MXene-based devices, besides laying the groundwork for engineering MXenes with tailored polyatomic surface terminations aimed at diverse technological applications, including high-performance catalysis, advanced energy storage systems, and nanoelectronics.

## Supplementary Information

Supplementary Information.

## Data Availability

The data used, generated, and/or analyzed during the current study are available from the corresponding authors on reasonable request.
