# Supplementary material for: Structural, electronic and thermodynamic properties of triatomic borate-terminated MXene surfaces
Source: Sci Rep. 2025 Sep 12;15:32513. doi: 10.1038/s41598-025-17851-z (PMC12432132; doi:10.1038/s41598-025-17851-z)
Supplement: Supplementary file 1 — Supplementary Information. [file 41598_2025_17851_MOESM1_ESM.pdf]

Supporting Information for

Structural, electronic and thermodynamic properties of triatomic borate-terminated MXene surfaces

Guilherme Ribeiro Portugal*[a], Johanna Rosen*[a]

[a] Materials Design, Department of Physics, Chemistry and Biology (IFM)

Linköping University, SE-58183, Linköping, Sweden

Thermodynamic Modeling

Adsorption free energy of Cl and BO_2 terminations

When using chemical potentials (μ) for the assessment of thermodynamic-related properties, establishing appropriate constraints to each particular μ is of extremely importance and allows us to understand how such properties may vary under distinct experimental conditions.

As discussed in the main text, for Cl terminations, such limits are straightforwardly obtained. The lower (more negative) chemical potential limit or, alternatively, the Cl-poor limit, is constrained by the adsorption free energy of chlorine terminations (ΔH_{ads}^{Cl}), which should be always negative so that competition between Cl and BO_2 terminations take place. By setting $\Delta H_{ads}^{Cl} = 0$ eV, we find that the poorest Cl condition in which the adsorption of Cl is still negative occurs when μ_{Cl} = -3.82 eV. On the other hand, the upper limit of μ_{Cl} is constrained by the stability of its precursor phase. According to the reported synthesis route and conditions, we consider that Cl^- ions which later become surface terminations on the MXene come from $CuCl_2$. This means that the chemical potentials of Cu (μ_{Cu}) and Cl will be constrained by the stability of the $CuCl_2$ phase as follows:

$$\mu_{CuCl_2} = \mu_{Cu} + 2\mu_{Cl} \quad , \tag{1}$$

where μ_{CuCl_2} is the chemical potential of $CuCl_2$, which in this case corresponds to the electronic enthalpy of solid $CuCl_2$ in its reference phase. The respective bounds to each individual chemical potential are governed by the formation of the elemental reference phase of each atomic species. In other words:

$$\mu_{Cu} \leq \mu_{Cu}^{metal} \quad , \tag{2}$$

$$\mu_{Cl} \leq \mu_{Cl}^{gas} \quad . \tag{3}$$

Now, combining equations 2 and 3 with equation 1, the lower bounds on μ_{Cu} and μ_{Cl} become the upper bounds for the corresponding species:

$$\mu_{CuCl_2} - 2\mu_{Cl}^{gas} \leq \mu_{Cu} \quad , \tag{4}$$

$$\mu_{CuCl_2} - \mu_{Cu}^{metal} \leq 2\mu_{Cl} \quad . \tag{5}$$

In order to avoid any misunderstanding related to the reference choice for each system, we include them explicitly as:

$$\Delta\mu_{Cu} = \mu_{Cu} - \mu_{Cu}^{metal} \quad , \tag{6}$$

$$\Delta\mu_{Cl} = \mu_{Cl} - \mu_{Cl}^{gas} \quad , \tag{7}$$

$$\Delta\mu_{CuCl_2} = \mu_{CuCl_2} - \mu_{Cu}^{metal} - 2\mu_{Cl}^{gas} = \Delta H_f^o(CuCl_2) \quad , \tag{8}$$

where $\Delta H_f^o(CuCl_2)$ is the standard formation enthalpy of $CuCl_2$ at 0K. Using such definitions, equation 1 becomes:

$$\Delta\mu_{CuCl_2} = \Delta\mu_{Cu} + 2\Delta\mu_{Cl} = \Delta H_f^o(CuCl_2) \quad . \tag{9}$$

A comprehensive plot of equation 9 is shown in Figure S11. Under Cl-rich conditions, we can take $\mu_{Cl} = \mu_{Cl}^{gas} = \frac{1}{2}H(Cl_2)$ (where $H(Cl_2)$ is the electronic enthalpy of a Cl_2 molecule) as the upper boundary for μ_{Cl}. Nonetheless, we also have to take into account the other extreme scenario, i.e., Cl-poor (or Cu-rich) conditions. In such a case, the chemical potential for copper, μ_{Cu} is taken from its most stable elemental metallic phase at 0K ($\mu_{Cu} = \mu_{Cu}^{metal}$). As a result, the chemical potential for chlorine might be as low as μ_{Cl} = -1.30 eV so that the stability of $CuCl_2$ can be maintained. It is noted that the upper limit obtained for μ_{Cl} under Cl-poor conditions is still higher than the lower limit established when setting $\Delta H_{ads}^{Cl} = 0$ eV. This indicates that considering $CuCl_2$ as the precursor for Cl terminations, their adsorption free energy will always be negative and hence the adsorption shall be thermodynamic spontaneous. To take into account the worst Cl scenario for which this is true, we take -1.30 eV as the upper limit for μ_{Cl} in the main text. That being settled, we can then write the full constraints for μ_{Cl} as:

$$-3.82 \text{ eV} \leq \mu_{Cl} \leq (-1.30 \text{ eV}, -0.35 \text{ eV}) \quad . \tag{10}$$

Now that the chemical potential for Cl terminations is defined, we move on for BO_2-terminated cases. The lower chemical potential limit corresponding to BO_2-poor conditions is again constrained by the adsorption free energy of metaborate terminations ($\Delta H_{ads}^{BO_2}$), which must be negative to ensure competition between Cl and BO_2 terminations. By setting $\Delta H_{ads}^{BO_2} = 0$ eV, we find that the poorest BO_2 condition in which the adsorption of BO_2 terminations is still negative occurs when μ_{BO_2} = -21.68 eV. Similarly to what was considered for chlorine species, the upper limit of μ_{BO_2} is constrained by the stability of its precursor phase. According to the reported synthesis route, BO_2^- ions come from the sodium metaborate ($NaBO_2$) generated during the decomposition of borax ($Na_2B_4O_7{\cdot}10H_2O$). Thus, we can rewrite equation 1 for $NaBO_2$ as a function of Na (μ_{Na}), B (μ_B) and O (μ_O) chemical potentials:

$$\mu_{NaBO_2} = \mu_{Na} + \mu_B + 2\mu_O \quad , \tag{11}$$

where μ_{NaBO_2} is the chemical potential of NaBO$_2$ (electronic enthalpy of its solid reference phase). The boundaries for each individual chemical potential we be given by:

$$\mu_{Na} \leq \mu_{Na}^{metal} \quad , \tag{12}$$

$$\mu_B \leq \mu_B^{metal} \quad , \tag{13}$$

$$\mu_O \leq \mu_O^{gas} \quad . \tag{14}$$

Setting up all the respective references, we hence write:

$$\Delta\mu_{Na} = \mu_{Na} - \mu_{Na}^{metal} \quad , \tag{15}$$

$$\Delta\mu_B = \mu_B - \mu_B^{metal} \quad , \tag{16}$$

$$\Delta\mu_O = \mu_O - \mu_O^{gas} \quad , \tag{17}$$

$$\Delta\mu_{NaBO_2} = \mu_{NaBO_2} - \mu_{Na}^{metal} - \mu_B^{metal} - \mu_O^{gas} = \Delta H_f^o(NaBO_2) \quad , \tag{18}$$

where $\Delta H_f^o(NaBO_2)$ is the standard formation enthalpy of NaBO$_2$ at 0K. Taking such definition into consideration equation 11 is written as:

$$\Delta\mu_{NaBO_2} = \Delta\mu_{Na} + \Delta\mu_B + 2\Delta\mu_O = \Delta H_f^o(NaBO_2) \quad . \tag{19}$$

However, given the instability and reactivity of BO$_2^-$ ions, it is reasonable to assume that metaborate terminations will likely to adsorb onto the MXene surface as intact units. We can thus define its chemical potential (μ_{BO_2}) under the same references as being:

$$\mu_{BO_2} = \mu_B + 2\mu_O \quad , \tag{20}$$

and, analogously, we also have:

$$\Delta\mu_{BO_2} = \mu_{BO_2} - \mu_B^{metal} - 2\mu_O^{gas} = \Delta H_f^o(BO_2) \quad , \tag{21}$$

$$\Delta\mu_{BO_2} = \Delta\mu_B + 2\Delta\mu_O = \Delta H_f^o(BO_2) \quad , \tag{22}$$

where $\Delta H_f^o(BO_2)$ is the standard formation enthalpy of BO$_2$ at 0K. Now, putting the relations described by equations 19 and 22 together results in:

$$\Delta\mu_{NaBO_2} = \Delta\mu_{Na} + \Delta\mu_{BO_2} = \Delta H_f^o(NaBO_2) \quad . \tag{23}$$

The only issue in this case is related to the fact that to establish definite values for μ_{BO_2} we need to know the reference chemical potential of metaborate units ($\mu^o_{BO_2}$), which is unfortunately unknown. A reasonable guess in this case is to take $\Delta H^o_f(BO_2) = 0$ eV, which would imply, according to equations 21 and 22 in $\mu^o_{BO_2} = \mu^{metal}_B + 2\mu^{gas}_O$. This results in a reference chemical potential for metaborate units of $\mu^o_{BO_2} =$ -12.13 eV, and allows us to proceed with the analysis as done for Cl-terminated MXenes. The plot for equation 23 can be seen in Figure S12. For BO_2-rich conditions, we take $\mu^o_{BO_2}$ as the upper limit for the chemical potential of BO_2 terminations. On the other hand, the BO_2-poor limit is obtained when $\mu_{Na} = \mu^{metal}_{Na}$ (Na-rich conditions). Consequently, μ_{BO_2} can be as low as -24.44 eV so that the stability of its $NaBO_2$ precursor is preserved. Yet, μ_{BO_2} = -24.44 eV is below the lower limit defined when making $\Delta H^{BO_2}_{ads} = 0$ eV. This is, in contrast with Cl terminations, an indicative that considering $NaBO_2$ as the precursor for metaborate ion, the adsorption of BO_2 terminations will not always be thermodynamic spontaneous. For that to be the case, the chemical potential of sodium must be at least $\mu_{Na} =$ -1.51 eV. As sodium metaborate originates from the thermal decomposition of borax, added to the fact the molten salt mixture will decreased the reactivity and lower the chemical potential of Na cations (*vide infra*), it is very likely that μ_{BO_2} will fall within the dark blue area in Figure S12. Finally, we are able to write the constraints for μ_{BO_2} as:

$$-21.68 \text{ eV} \leq \mu_{BO_2} \leq -12.13 \text{ eV} \quad . \tag{24}$$

In a system where sodium is incorporated into a stable compound such as $NaBO_2$, the chemical potential of sodium will be significantly lower (more negative) compared to its elemental reference phase (pure metallic sodium). Furthermore, the synthesis follows a molten salt protocol including other compounds like $CuCl_2$, NaCl, and KCl at high temperature. While such an environment can facilitate ionic transport and potentially alter the equilibrium, it stabilizes ions by lowering the free energy of ionic species, thereby reducing their chemical potentials. In essence, the molten salt bath acts like a stabilizing medium that lowers the chemical potential of species dissolved within it—particularly ions—compared to their pure elemental phases. The elemental reference phase of sodium is pure Na metal, in which sodium atoms are not bound to oxygen or boron. In that state, the sodium chemical potential is relatively higher. In contrast, as soon as sodium becomes part of a strongly bonded ionic lattice, such as $NaBO_2$, its chemical potential drops. Within $NaBO_2$, especially in a high-temperature molten salt environment, the chemical potential of sodium is much lower than in its elemental metallic form. Therefore, it is reasonable to admit that μ_{BO_2} will be higher than -21.68 eV and fall within the dark blue shaded area in Figure S12, which allows the spontaneous adsorption of BO_2 terminations.

We can finally plot such limits together with the adsorption free energy of each termination, as displayed in Figure S13. Nevertheless, the only direct conclusion we can draw is that there will be competition for spontaneous adsorption between Cl and BO_2 terminations within the established range of chemical potentials. But how can we infer which termination is more likely to be preferred? One possible argument is that the minimum adsorption energy for the Cl terminations, naturally obtained under Cl-rich conditions, is -2.52 eV. In contrast, for BO_2 terminations, the same adsorption energy is achieved when μ_{BO_2} = -19.15 eV, which is about 7.0 eV lower than its reference upper limit of -12.13 eV. This indicates that the adsorption energy of the BO_2 units can be significantly lower and thus more favorable than the theoretical limit for Cl terminations. For instance, if

we take μ_{BO_2} = -15.0 eV, an intermediate value, the corresponding adsorption energy is -6.68 eV. Therefore, within the approximations made herein, we can understand why the formation of the BO_2-terminated surface is significantly more favorable than that of the Cl-terminated one in such an environment. Another possible explanation is that due to the created molten salt environment the chemical potential of Cl ions might be too low to enable spontaneous adsorption under the experimental conditions. In this case, there would be no competition, and the μ_{BO_2} terminations would always be favored.

Now that we have established the thermodynamic modeling using $Ti_3C_2T_x$ as an example, we extend this approach to other MXene structures of interest. Assuming that the experimental conditions remain consistent, with only slight adjustments in temperature based on the precursor phase (as described in the main paper), the upper limits for the μ_{Cl} and μ_{BO_2} will remain unchanged. However, the lower limits will vary, as they are constrained by the adsorption free (ΔH_{ads}) specific to each termination species and MXene structure. Table S11 compiles the chemical potential ranges for all the investigated MXene structures.

Alternative chemical reaction-based approach

As experimentally reported, EDX, XPS, and FTIR analyses confirmed the absence of both OH and Cl terminations in the synthesized BO_2-MXenes, thus indicating that their surfaces are exclusively covered by BO_2 functional groups. Such a fact is particularly is interesting because the molten salt-based synthesis route (performed at ∼800°C) includes $CuCl_2$, NaCl, and KCl, which would normally favor Cl-terminated MXenes if borax were not included. Therefore, the fundamental question is raised: why do BO_2-capped surfaces ultimately prevail? To address this issue, one could employ DFT calculations to evaluate the formation of both BO_2- and Cl-terminated MXenes via the following reactions:

$$2M_{n+1}Al_nC_2 + 3CuCl_2 \rightarrow 2M_{n+1}X_n + 2AlCl_3 + 3Cu \quad , \tag{25}$$

$$Na_2B_4O_7 \rightarrow 2NaBO_2 + B_2O_3 \quad , \tag{26}$$

$$M_{n+1}X_n + CuCl_2 + 2NaBO_2 \rightarrow M_{n+1}X_n(BO_2)_2 + 2NaCl + Cu \quad , \tag{27}$$

where M and X represent the transition metal and the carbon/nitrogen sites in MAX or MXene phases, respectively, and n = 1–3. Within this synthesis route, reaction 25 describes the usual molten salt exfoliation, reaction 26 expresses the thermal decomposition of borax ($Na_2B_4O_7{\cdot}10H_2O$) into sodium metaborate ($NaBO_2$) and boron trioxide (B_2O_3), and reaction 27 represents how the bare MXene is capped by $-BO_2$ groups. If, on the other hand, Cl^- were the preferred termination species, reaction 27 would be replaced by

$$M_{n+1}X_n + CuCl_2 + 2NaBO_2 \rightarrow M_{n+1}X_nCl_2 + 2NaBO_2 + Cu \quad . \tag{28}$$

By comparing the total energy summation for the right-hand sides of reactions 27 and 28 (excluding Cu, which cancels out on both sides), D. Li *et al.*, *Nat. Mater.* **23**, 1085–1092 (2024) evaluated what has been claimed to be the thermodynamic stability of Cl and BO_2 terminations. Using DFT total energies for each compound, the total energy summation reported were of -109.0 eV for reaction 27 and -106.1 eV for reaction 28. Based on that, it was concluded that BO_2-capped surfaces exhibit slightly higher stability.

Supplementary Figures

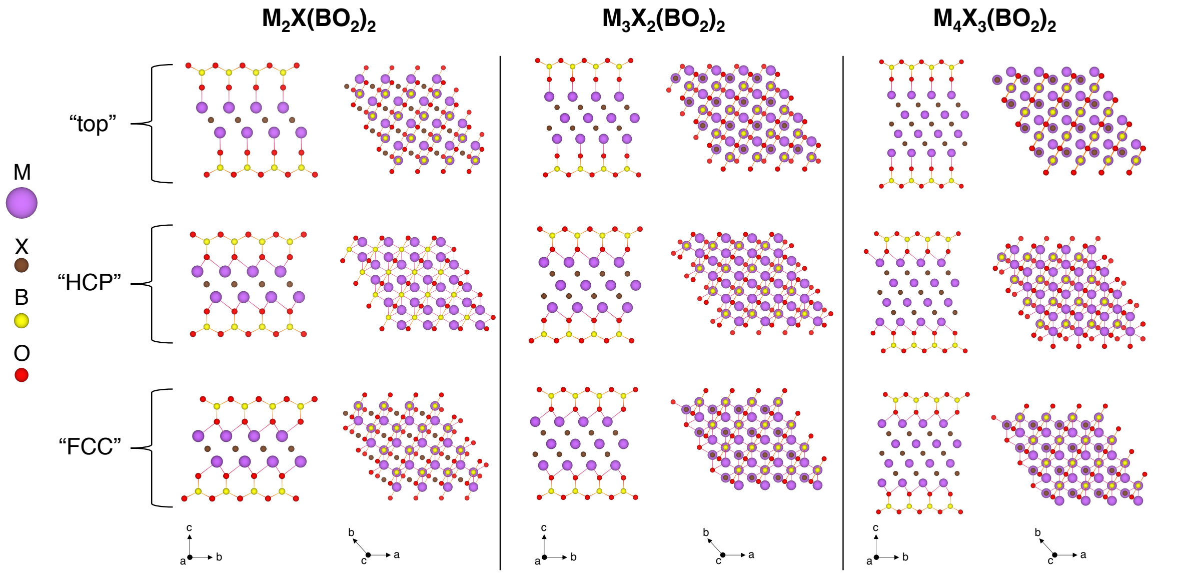

Figure S1: Possible adsorption sites for BO_2 terminations onto MXene surfaces. The figure presents the structural configurations for M_2X (left panel), M_3X_2 (central panel), and M_4X_3 (right panel) MXene families, illustrating the three possible termination sites: the "top" site, where termination species are located directly above the surface M atoms; the hollow "HCP" site, corresponding to terminations above X atoms; and the hollow "FCC" site, where termination species are positioned over M atoms in the inner layers. Each structure is depicted along different crystallographic orientations, frontal and top views, showing the arrangement of BO_2 terminations relative to the transition-metal sublattice.

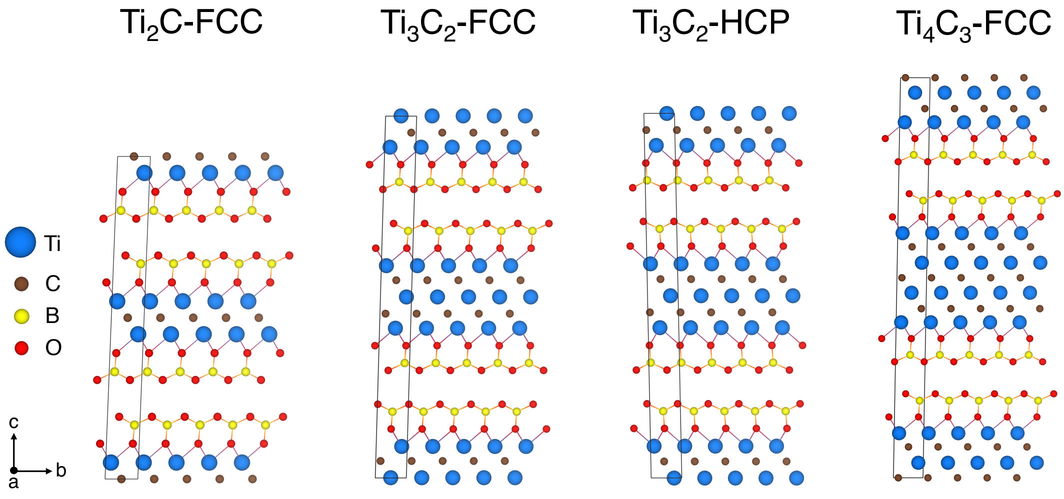

Figure S2: Relaxed structures of Ti-based MXenes. Both FCC and HCP sites are shown for Ti_3C_2.

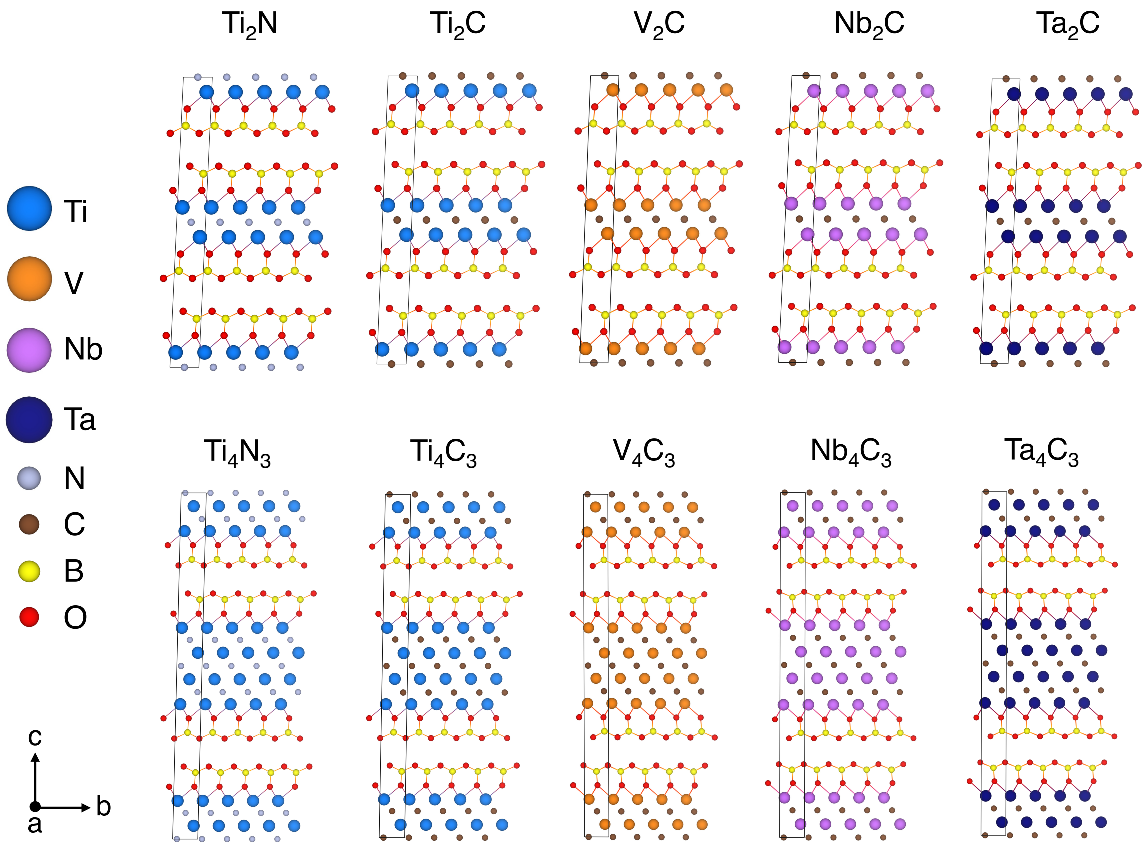

Figure S3: Relaxed structures of all simulated BO_2-terminated M_2X (top panel) and M_4X_3 (bottom panel) MXenes. In M_2X and Ti-based M_4X_3 MXenes, BO_2 terminations occupy the FCC site on the surface, while in other M_4X_3 MXenes these terminations are located at the HCP sites.

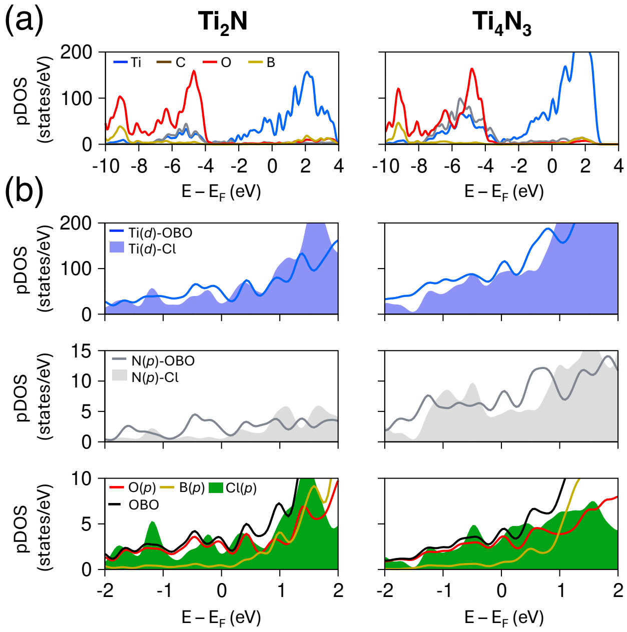

Figure S4: Projected density of states (pDOS) for Ti_2N (left panels) and Ti_4N_3 (right panels). (a) pDOS for BO_2-terminated MXenes. (b) Close-up comparison of the pDOS for each atomic species in BO_2- and Cl-terminated MXenes, where the shaded areas represent the Cl-terminated systems and the standard lines correspond to the BO_2-terminated systems. For simplicity, OBO here stands for BO_2.

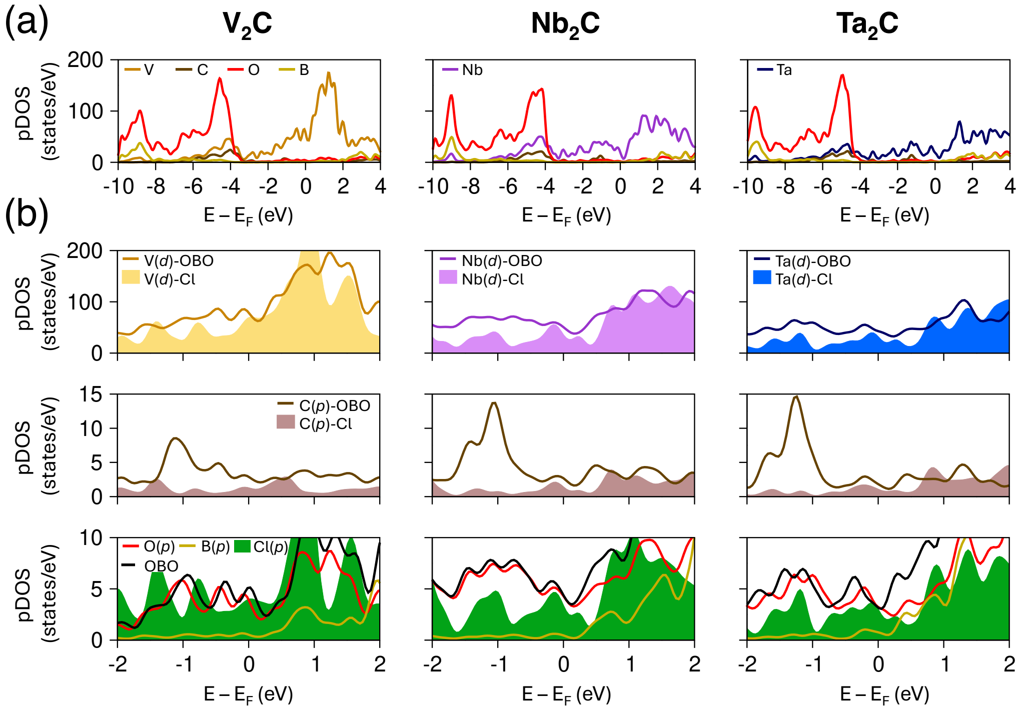

Figure S5: Projected density of states (pDOS) for V_2C (left panels), Nb_2C (central panels), and Ta_2C (right panels). (a) pDOS for BO_2-terminated MXenes. (b) Close-up comparison of the pDOS for each atomic species in BO_2- and Cl-terminated MXenes, where the shaded areas represent the Cl-terminated systems and the standard lines correspond to the BO_2-terminated systems. For simplicity, OBO here stands for BO_2.

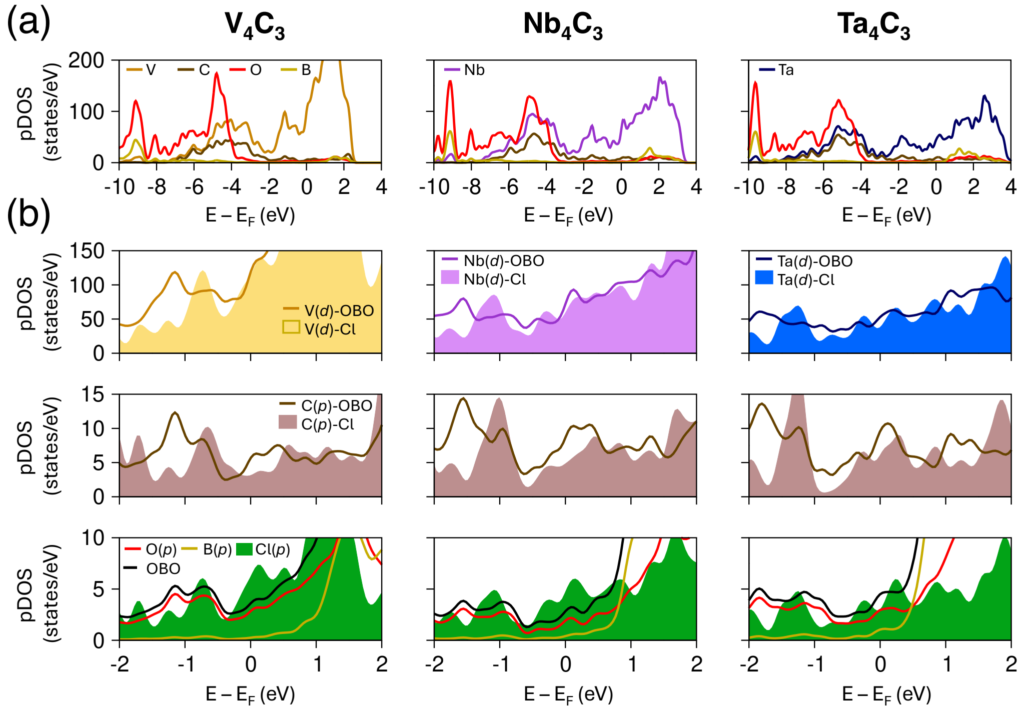

Figure S6: Projected density of states (pDOS) for V_4C_3 (left panels), Nb_4C_3 (central panels), and Ta_4C_3 (right panels). (a) pDOS for BO_2-terminated MXenes. (b) Close-up comparison of the pDOS for each atomic species in BO_2- and Cl-terminated MXenes, where the shaded areas represent the Cl-terminated systems and the standard lines correspond to the BO_2-terminated systems. For simplicity, OBO here stands for BO_2.

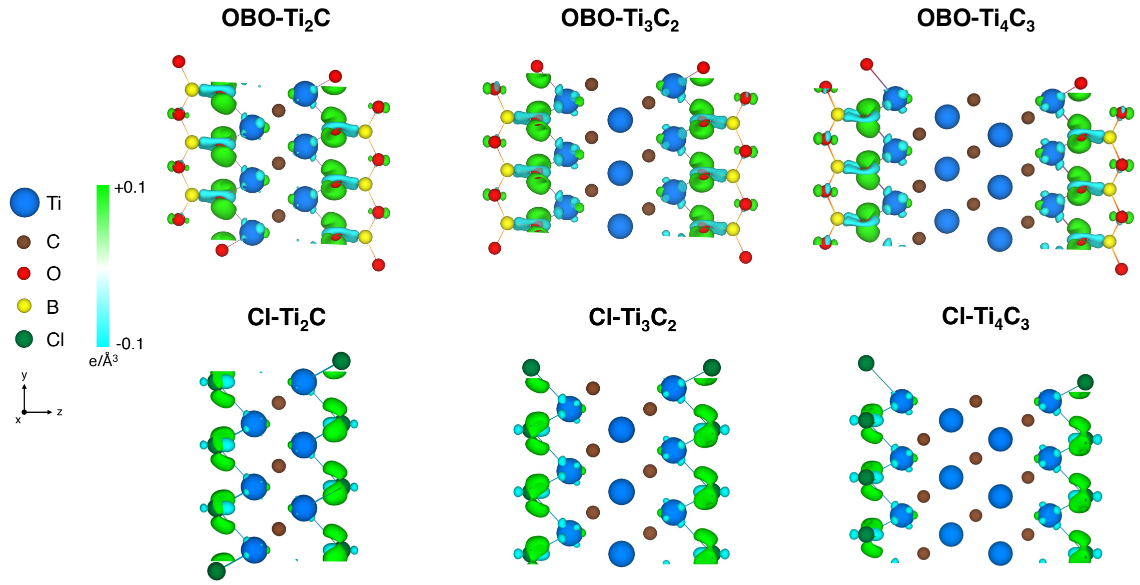

Figure S7: Three dimensional plots of the charge density difference (CDD) comparing BO_2-terminated MXenes (top panels) with Cl-terminated MXenes (bottom panels). Isosurface value $= \pm$ 0.01 e/Å. For simplicity, OBO here stands for BO_2.

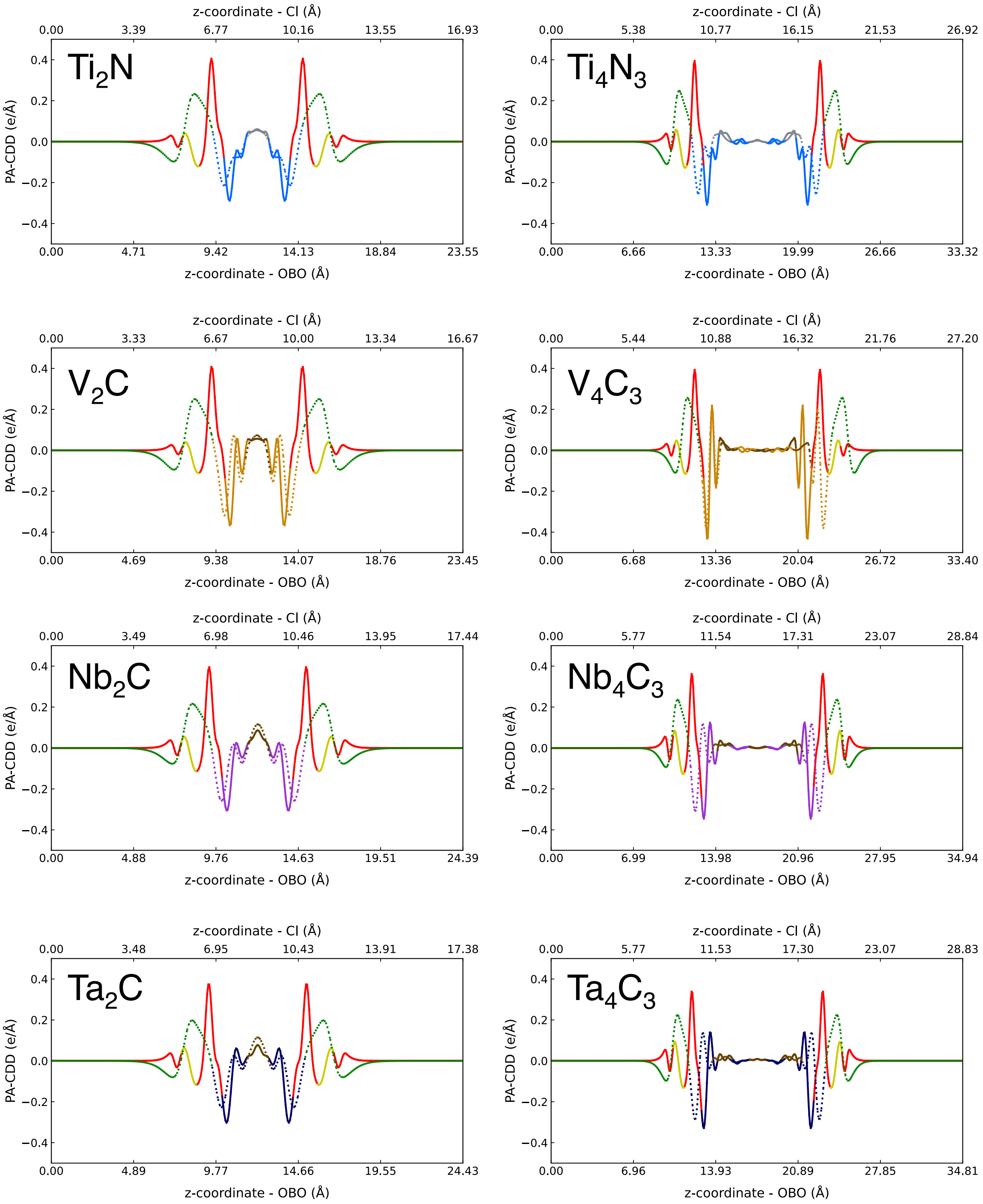

Figure S8: Planar-averaged charge density difference (PA-CDD) along the out-of-plane (z) direction, comparing BO_2 (solid line) and Cl (dotted line) terminations. M_2X (M_4X_3) MXenes are shown in the left (right) panel. Colors along each line indicate the atomic planes encountered at each z-coordinate: green for Cl, blue for Ti, orange for V, purple for Nb, dark blue for Ta, red for O, golden for B, gray for N, and brown for C. For simplicity, OBO here stands for BO_2.

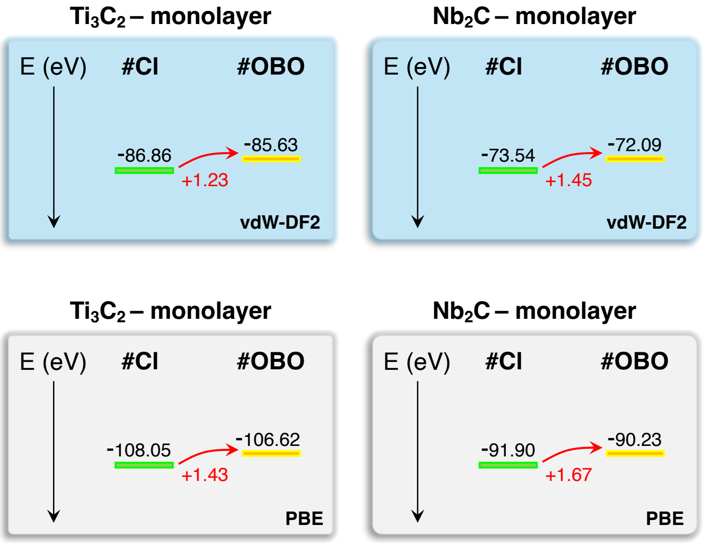

Figure S9: Schematics for the "thermodynamic stability" of monolayered $Ti_3C_2(BO_2)_2$ (left panels) and $Nb_2C(BO_2)_2$ (right panels) achieved using the chemical reactions approach (comparison between the right-hand side of reactions 27 and 28. It is noted that, for both PBE (bottom gray panels) and rev-vdW-DF2 (top blue panels) functionals, such a method always predicts higher "thermodynamic stability" of Cl-terminated MXenes when compared to BO_2-MXenes. For simplicity, OBO here stands for BO_2.

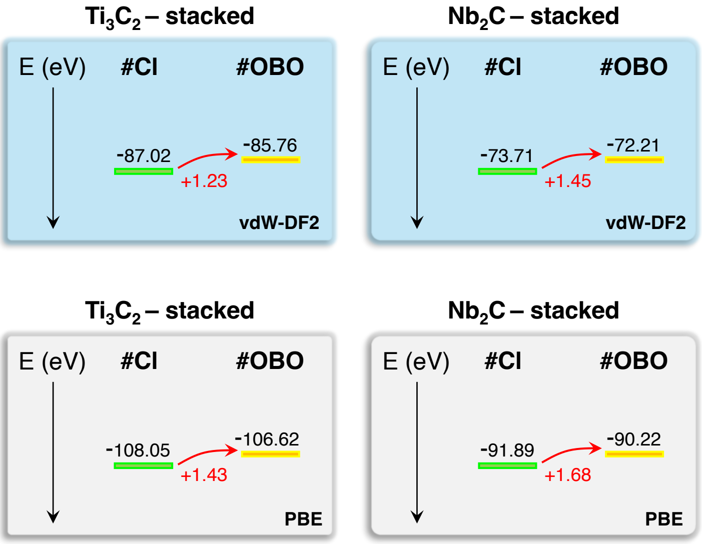

Figure S10: Schematics for the "thermodynamic stability" of multilayered $Ti_3C_2(BO_2)_2$ (left panels) and $Nb_2C(BO_2)_2$ (right panels) achieved using the chemical reactions approach. It is noted that, for both PBE (bottom gray panels) and rev-vdW-DF2 (top blue panels) functionals, such a method always predicts higher "thermodynamic stability" of Cl-terminated MXenes when compared to BO_2-MXenes. When using PBE, the values are basically the same for mono or multilayered MXenes, which arises from the fact the PBE is not able to capture the correct physics behind the interaction of stacked MXene layers that occur mainly through dispersive van der Waals forces. For simplicity, OBO here stands for BO_2.

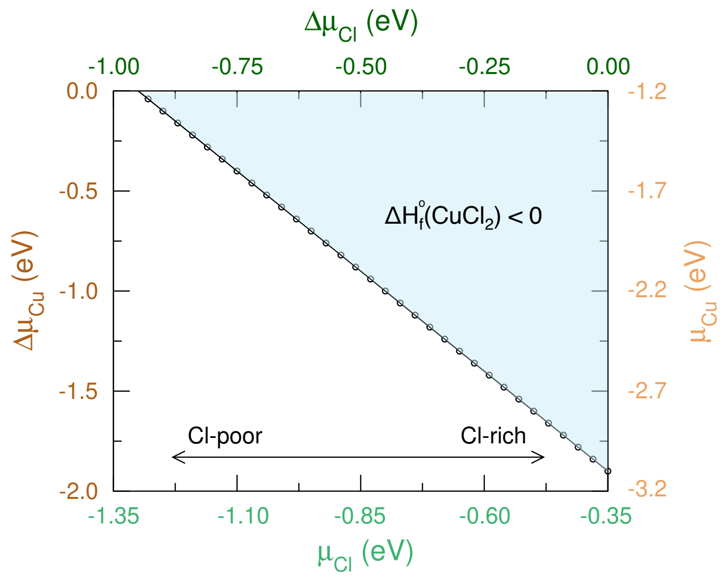

Figure S11: Calculated stability region for $CuCl_2$ formation as a function of copper and chlorine chemical potentials. The shaded area indicates conditions where the standard formation enthalpy $\Delta H_f^o(CuCl_2)$ is negative, highlighting the thermodynamic stability region. Moving horizontally from left to right corresponds to transitioning from Cl-poor (Cu-rich) to Cl-rich (Cu-poor) conditions.

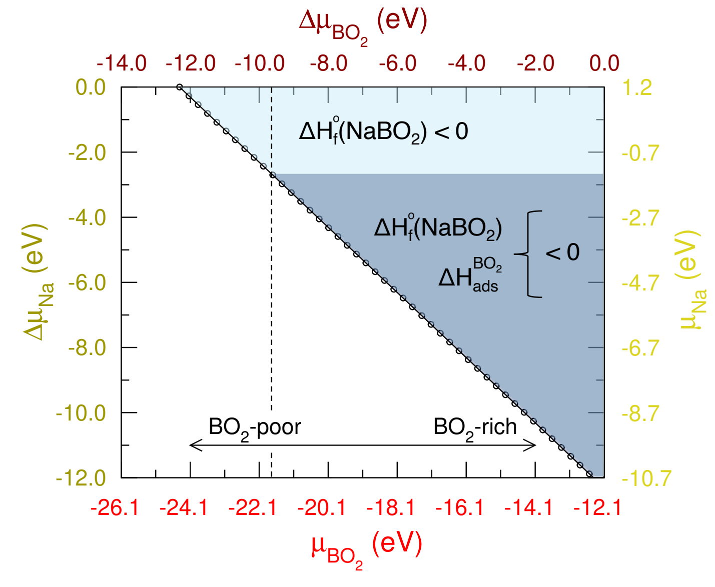

Figure S12: Calculated stability region for $NaBO_2$ formation as a function of sodium and metaborate chemical potentials. The area shaded in dark blue indicates conditions where the standard formation enthalpy $\Delta H_f^o(NaBO_2)$ and the adsorption free energy for metaborate termination $\Delta H_{ads}^{BO_2}$ are both negative. The later limit is also represented by the dashed vertical line at μ_{BO_2} = -21.68 eV. The area shaded in light blue, which includes the whole dark blue area and extends up to the origin, highlights the thermodynamic stability region of the precursor phase. Moving horizontally from left to right corresponds to transitioning from BO_2-poor (Na-rich) to BO_2-rich (Na-poor) conditions.

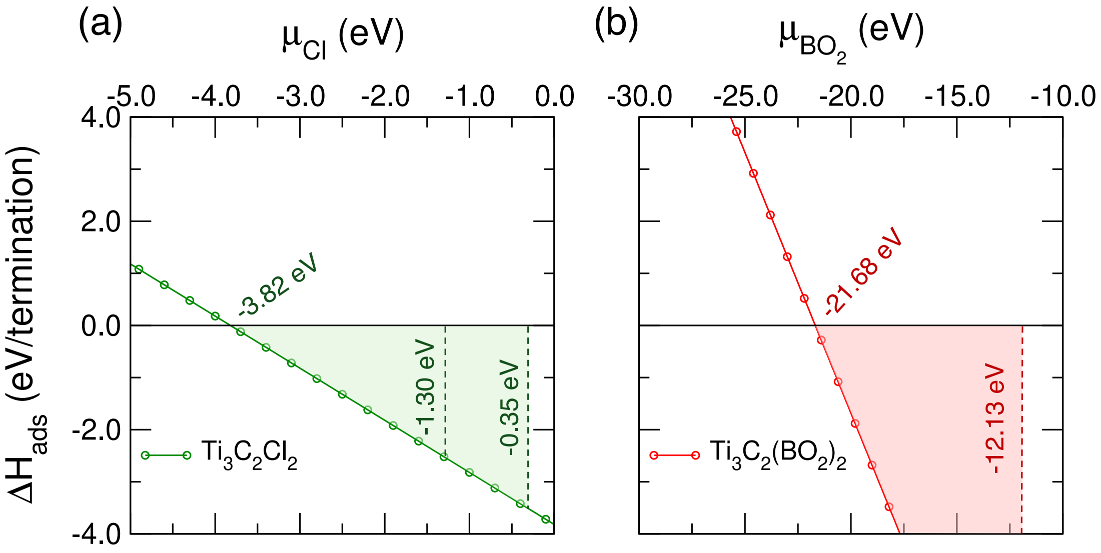

Figure S13: Adsorption free energy (ΔH_{ads}) as a function of the chemical potential for (a) chlorine (μ_{Cl}) and (b) metaborate (μ_{BO_2}) terminations. The shaded regions correspond to the stability ranges of μ_{Cl} and μ_{BO_2}. In (a), the lower limit of -3.82 eV and upper limits of -1.30 eV and -0.35 eV for μ_{Cl} are displayed, whereas in (b) the lower limit for μ_{BO_2} is marked at -21.68 eV, and its upper limit at -12.13 eV.

Supplementary Tables

Table S1: Optimized lattice parameters of multilayered Cl-terminated MXenes. For each compound, the energetically preferred BO_2 adsorption site (FCC or HCP) is reported together with their respective in-plane ($a = b$) and out-of-plane (c) lattice constants, and the three lattice angles α, β, and γ.

MXene	Cl site	$a = b$ (Å)	c (Å)	α=β (°)	γ (°)
Ti_2N	FCC	3.21	16.93	90.00	120.00
Ti_2C	FCC	3.21	17.20	90.00	120.00
V_2C	FCC	3.16	16.67	90.00	120.00
Nb_2C	FCC	3.34	17.50	90.00	120.00
Ta_2C	FCC	3.33	17.44	90.00	120.00
Ti_3C_2	FCC	3.17	22.13	90.00	120.00
Ti_4N_3	FCC	3.09	26.98	90.00	120.00
Ti_4C_3	FCC	3.15	27.13	90.00	120.00
V_4C_3	HCP	2.98	27.27	90.00	120.00
Nb_4C_3	HCP	3.18	28.91	90.00	120.00
Ta_4C_3	HCP	3.15	28.90	90.00	120.00

Table S2: Relative lattice constants and angles of each BO_2-terminated MXene with respect to their parent MAX phases.

MXene	BO_2 site	$a = b$ (Å)	c (Å)	α(°)	β (°)	γ (°)
Ti_2N	FCC	0.04	9.90	-5.64	5.64	9.68
Ti_2C	FCC	-0.02	9.72	-4.64	4.64	8.61
V_2C	FCC	0.09	10.32	-4.59	4.59	9.75
Nb_2C	FCC	0.04	10.52	-5.82	5.82	10.88
Ta_2C	FCC	0.05	10.53	-5.16	5.16	10.36
Ti_3C_2	FCC	-0.03	9.78	-3.28	3.28	7.01
Ti_4N_3	FCC	0.01	9.91	-2.77	2.77	6.15
Ti_4C_3	FCC	-0.04	9.82	-1.99	1.99	5.85
V_4C_3	HCP	-0.03	10.67	0.35	-0.35	4.85
Nb_4C_3	HCP	-0.05	10.62	-0.32	0.32	5.61
Ta_4C_3	HCP	-0.06	10.60	-0.33	0.33	5.08

Table S3: Relative lattice constants and angles of each Cl-terminated MXene with respect to their parent MAX phases.

MXene	Cl site	$a = b$ (Å)	c (Å)	$\alpha(°)$	β (°)	γ (°)
Ti_2N	FCC	0.22	3.28	-0.10	0.00	0.00
Ti_2C	FCC	0.14	3.46	-0.10	0.00	0.00
V_2C	FCC	0.25	3.54	-0.10	0.00	0.00
Nb_2C	FCC	0.21	3.57	0.00	0.00	0.00
Ta_2C	FCC	0.23	3.48	-0.09	0.00	0.00
Ti_3C_2	FCC	0.09	3.48	0.00	0.00	0.00
Ti_4N_3	FCC	0.10	3.50	0.00	0.00	0.00
Ti_4C_3	FCC	0.07	3.53	0.00	0.00	0.00
V_4C_3	HCP	0.06	4.47	0.00	0.00	0.00
Nb_4C_3	HCP	0.02	4.53	0.00	0.00	0.00
Ta_4C_3	HCP	0.02	4.62	0.00	0.00	0.00

Table S4: Layer thickness of all calculated MXenes and MAX phases. The layer thickness (t) is defined as the vertical distance between opposing outermost M (metal) atomic planes. The relative variation in thickness (Rel. T = BO_2 and Rel. T = Cl) is obtained subtracting the thickness of the MAX phase from the respective MXene thickness. All values are given in Å.

MXene Phase	MAX Phase	MAX	T = BO_2	T = Cl	Rel. T = BO_2	Rel. T = Cl
Ti_2NT_2	Ti_2AlN	2.34	2.41	2.04	0.07	-0.29
Ti_2CT_2	Ti_2AlC	2.29	2.39	2.19	0.10	-0.10
V_2CT_2	V_2AlC	2.24	2.32	1.92	0.08	-0.32
Nb_2CT_2	Nb_2AlC	2.49	2.60	2.14	0.12	-0.35
Ta_2CT_2	Ta_2AlC	2.51	2.60	2.09	0.09	-0.42
$Ti_3C_2T_2$	Ti_3AlC_2	4.74	4.90	4.59	0.16	-0.16
$Ti_4N_3T_2$	Ti_4AlN_3	7.25	7.36	6.95	0.11	-0.31
$Ti_4C_3T_2$	Ti_4AlC_3	7.22	7.44	7.05	0.21	-0.17
$V_4C_3T_2$	V_4AlC_3	7.04	7.37	7.01	0.32	-0.04
$Nb_4C_3T_2$	Nb_4AlC_3	7.67	8.07	7.67	0.40	0.01
$Ta_4C_3T_2$	Ta_4AlC_3	7.64	8.01	7.65	0.37	0.02

Table S5: Preferred BO_2 adsorption site and the thickness of BO_2 termination layer (t-BO_2) of all calculated BO_2-terminated MXenes in their multilayered stacked structure lowest energy configuration.

MXene	Site	t-BO_2 (Å)
Ti_2N	FCC	3.32
Ti_2C	FCC	3.32
V_2C	FCC	3.34
Nb_2C	FCC	3.45
Ta_2C	FCC	3.48
Ti_3C_2	FCC	3.31
Ti_4N_3	FCC	3.31
Ti_4C_3	FCC	3.29
V_4C_3	HCP	3.31
Nb_4C_3	HCP	3.40
Ta_4C_3	HCP	3.40

Table S6: Bader charge analysis for BO_2- and Cl-terminated Ti_2N and Ti_2C MXenes. The Bader charges (in electrons) are reported for different atomic species: O-T represents oxygen atoms bonded to Ti, and O-S corresponds to the outermost oxygen atoms on the BO_2-terminated surface. Negative values reflect electron accumulation, while positive values indicate electron depletion.

Ti_2N			Ti_2C		
Atom	BO_2	Cl_2	Atom	BO_2	Cl_2
Ti1	1.31	1.09	Ti1	1.12	1.07
Ti2	1.12	1.03	Ti2	1.25	0.93
N1	-1.20	-1.27	C1	-1.11	-1.16
O1-T	-1.17	-	O1-T	-1.20	-
O2-T	-1.19	-	O2-T	-1.26	-
O3-S	-1.35	-	O3-S	-1.37	-
O4-S	-1.36	-	O4-S	-1.42	-
B1	1.87	-	B1	1.97	-
B2	1.95	-	B2	2.02	-
Cl1	-	-0.45	Cl1	-	-0.46
Cl2	-	-0.40	Cl2	-	-0.38

Table S7: Bader charge analysis for BO_2- and Cl-terminated V_2N, Nb_2C, and Ta_2C MXenes. The Bader charges (in electrons) are reported for different atomic species: O-T represents oxygen atoms bonded to V, Nb, or Ta as terminations, and O-S corresponds to the outermost oxygen atoms on the BO_2-terminated surface. Negative values reflect electron accumulation, while positive values indicate electron depletion.

V_2C			Nb_2C			Ta_2C		
Atom	BO_2	Cl_2	Atom	BO_2	Cl_2	Atom	BO_2	Cl_2
V1	1.32	0.87	Nb1	1.54	1.46	Ta1	1.21	1.57
V2	1.23	0.85	Nb2	1.40	1.43	Ta2	1.59	1.58
C1	-1.26	-1.03	C1	-1.52	-1.80	C1	-1.36	-2.03
O1-T	-1.40	-	O1-T	-1.31	-	O1-T	-1.16	-
O2-T	-1.33	-	O2-T	-1.22	-	O2-T	-1.19	-
O3-S	-1.40	-	O3-S	-1.36	-	O3-S	-1.32	-
O4-S	-1.37	-	O4-S	-1.40	-	O4-S	-1.26	-
B1	2.14	-	B1	1.94	-	B1	1.83	-
B2	2.07	-	B2	1.94	-	B2	1.67	-
Cl1	-	-0.36	Cl1	-	-0.55	Cl1	-	-0.56
Cl2	-	-0.33	Cl2	-	-0.54	Cl2	-	-0.57

Table S8: Bader charge analysis for BO_2-Ti_3C_2 and Cl-Ti_3C_2 MXenes. The Bader charges (in electrons) are reported for different atomic species: Ti-T refers to titanium atoms directly bonded to surface terminations, while Ti-C denotes central titanium atoms. O-T represents oxygen atoms bonded to Ti, and O-S corresponds to the outermost oxygen atoms on the BO_2-terminated surface. Negative values reflect electron accumulation, while positive values indicate electron depletion.

	Ti_3C_2	
Atom	BO_2	Cl_2
Ti1-T	1.47	1.19
Ti2-C	1.27	1.14
Ti3-T	1.24	1.15
C1	-1.41	-1.29
C2	-1.26	-1.27
O1-T	-1.32	-
O2-T	-1.31	-
O3-S	-1.38	-
O4-S	-1.27	-
B1	2.02	-
B2	1.95	-
Cl1	-	-0.46
Cl2	-	-0.45

Table S9: Bader charge analysis for BO_2- and Cl-terminated Ti_4N_3 and Ti_4C_3 MXenes. The Bader charges (in electrons) are reported for different atomic species: Ti-T refers to titanium atoms directly bonded to surface terminations, while Ti-C denotes central titanium atoms. O-T represents oxygen atoms bonded to Ti, and O-S corresponds to the outermost oxygen atoms on the BO_2-terminated surface. Negative values reflect electron accumulation, while positive values indicate electron depletion.

Ti_4N_3			Ti_4C_3		
Atom	BO_2	Cl_2	Atom	BO_2	Cl_2
Ti1-T	1.36	1.63	Ti1-T	1.45	1.26
Ti2-C	1.50	1.74	Ti2-C	1.34	1.33
Ti3-C	1.33	1.74	Ti3-C	1.23	1.12
Ti4-T	1.39	1.63	Ti4-T	1.29	1.21
N1	-1.46	-1.84	C1	-1.45	-1.44
N2	-1.47	-1.75	C2	-1.33	-1.28
N3	-1.34	-1.84	C3	-1.23	-1.24
O1-T	-1.32	-	O1-T	-1.33	-
O2-T	-1.30	-	O2-T	-1.29	-
O3-S	-1.33	-	O3-S	-1.39	-
O4-S	-1.31	-	O4-S	-1.38	-
B1	2.01	-	B1	2.05	-
B2	1.94	-	B2	2.03	-
Cl1	-	-0.66	Cl1	-	-0.48
Cl2	-	-0.66	Cl2	-	-0.49

Table S10: Bader charge analysis for BO_2- and Cl-terminated V_4C_3, Nb_4C_3 , and Ta_4C_3 MXenes. The Bader charges (in electrons) are reported for different atomic species:(V,Nb,Ta)-T refers to metals directly bonded to surface terminations, while (V,Nb,Ta)-C denotes central metal atoms. O-T represents oxygen atoms bonded to metals as terminations, and O-S corresponds to the outermost oxygen atoms on the BO_2-terminated surface. Negative values reflect electron accumulation, while positive values indicate electron depletion.

V_4C_3			Nb_4C_3			Ta_4C_3		
Atom	BO_2	Cl_2	Atom	BO_2	Cl_2	Atom	BO_2	Cl_2
V1-T	1.23	0.90	Nb1-T	1.52	1.49	Ta1-T	1.78	1.63
V2-C	1.15	1.24	Nb2-C	1.67	1.71	Ta2-C	1.81	1.95
V3-C	0.67	1.17	Nb3-C	1.46	1.71	Ta3-C	0.75	1.94
V4-T	1.20	0.94	Nb4-T	1.57	1.49	Ta4-T	1.67	1.63
C1	-1.13	-1.17	C1	-1.56	-1.74	C1	-1.81	-1.96
C2	-0.95	-1.19	C2	-1.68	-1.74	C2	-1.33	-1.98
C3	-0.92	-1.17	C3	-1.51	-1.74	C3	-1.30	-1.95
O1-T	-1.15	-	O1-T	-1.32	-	O1-T	-1.20	-
O2-T	-1.17	-	O2-T	-1.34	-	O2-T	-1.28	-
O3-S	-1.24	-	O3-S	-1.23	-	O3-S	-1.03	-
O4-S	-1.31	-	O4-S	-1.25	-	O4-S	-1.08	-
B1	1.77	-	B1	1.82	-	B1	1.42	-
B2	1.86	-	B2	1.84	-	B2	1.60	-
Cl1	-	-0.36	Cl1	-	-0.59	Cl1	-	-0.63
Cl2	-	-0.36	Cl2	-	-0.59	Cl2	-	-0.63

Table S11: Lower boundaries for μ_{Cl} and μ_{BO_2} achieved when the respective ΔH_{ads} is set to zero for different MXenes. The evaluated chemical potentials should therefore be bigger (less negative) then the values displayed so that the adsorption is energetically spontaneous for both terminations and competition is guaranteed. Smaller values (more negative) fall within a region too poor of the respective chemical potential and the adsorption becomes energetically unfavorable.

MXene	Site	μ_{BO_2} (eV)	μ_{Cl} (eV)
Ti_2N	FCC	-22.11	-3.75
Ti_2C	FCC	-22.08	-3.91
V_2C	FCC	-21.59	-3.18
Nb_2C	FCC	-21.20	-3.45
Ta_2C	FCC	-21.07	-3.29
Ti_3C_2	FCC	-21.68	-3.82
Ti_4N_3	FCC	-21.61	-3.48
Ti_4C_3	FCC	-21.48	-3.77
V_4C_3	HCP	-21.20	-2.76
Nb_4C_3	HCP	-20.45	-3.25
Ta_4C_3	HCP	-20.44	-3.14

Table S12: Calculated energy differences (ΔE) for the reactions behind the formation of Cl-terminated and BO_2-MXenes. The values correspond to different MXene compositions and termination sites (FCC or HCP) calculated using the rev-vdW-DF2 functional. Positive ΔE values indicate that the formation of BO_2-terminated MXenes is energetically unfavorable based on this model.

MXene	Site	$M_{n+1}X_nCl_2$ + 2$NaBO_2$ (eV)	$M_{n+1}X_n(BO_2)_2$ + 2NaCl (eV)	ΔE (eV)
Ti_2N	FCC	-71.27	-71.49	+0.22
Ti_2C	FCC	-70.53	-71.13	+0.60
V_2C	FCC	-70.10	-70.23	+0.13
Nb_2C	FCC	-72.09	-73.55	+1.45
Ta_2C	FCC	-75.22	-76.61	+1.38
Ti_3C_2	FCC	-85.63	-86.86	+1.23
	HCP	-85.36	-86.32	+0.96
Ti_4N_3	FCC	-103.33	-104.01	+0.68
	HCP	-103.52	-103.91	+0.39
Ti_4C_3	FCC	-102.44	-100.90	+1.54
	HCP	-101.97	-100.63	+1.34
V_4C_3	FCC	-100.95	-101.01	+0.06
	HCP	-100.95	-101.02	+0.07
Nb_4C_3	FCC	-104.96	-107.84	+2.89
	HCP	-105.46	-108.02	+2.56
Ta_4C_3	FCC	-112.59	-114.73	+2.14
	HCP	-112.59	-114.94	+2.36
